# Strain Interactions as a Mechanism for Dominant Strain Alternation and Incidence Oscillation in Infectious Diseases: Seasonal Influenza as a Case Study

**DOI:** 10.1371/journal.pone.0142170

**Published:** 2015-11-12

**Authors:** Xu-Sheng Zhang

**Affiliations:** 1 Modelling and Economics Unit, Department of Statistics, Modelling and Economics, Centre for Infectious Disease Surveillance and Control, Public Health England, London, United Kingdom; 2 Medical Research Council Centre for Outbreak Analysis and Modelling, Department of Infectious Disease Epidemiology, Imperial College School of Public Health, London, United Kingdom; University of Malaya, MALAYSIA

## Abstract

**Background:**

Many human infectious diseases are caused by pathogens that have multiple strains and show oscillation in infection incidence and alternation of dominant strains which together are referred to as epidemic cycling. Understanding the underlying mechanisms of epidemic cycling is essential for forecasting outbreaks of epidemics and therefore important for public health planning. Current theoretical effort is mainly focused on the factors that are extrinsic to the pathogens themselves (“extrinsic factors”) such as environmental variation and seasonal change in human behaviours and susceptibility. Nevertheless, co-circulation of different strains of a pathogen was usually observed and thus strains interact with one another within concurrent infection and during sequential infection. The existence of these intrinsic factors is common and may be involved in the generation of epidemic cycling of multi-strain pathogens.

**Methods and Findings:**

To explore the mechanisms that are intrinsic to the pathogens themselves (“intrinsic factors”) for epidemic cycling, we consider a multi-strain SIRS model including cross-immunity and infectivity enhancement and use seasonal influenza as an example to parameterize the model. The Kullback-Leibler information distance was calculated to measure the match between the model outputs and the typical features of seasonal flu (an outbreak duration of 11 weeks and an annual attack rate of 15%). Results show that interactions among strains can generate seasonal influenza with these characteristic features, provided that: the infectivity of a single strain within concurrent infection is enhanced 2−7 times that within a single infection; cross-immunity as a result of past infection is 0.5–0.8 and lasts 2–9 years; while other parameters are within their widely accepted ranges (such as a 2–3 day infectious period and the basic reproductive number of 1.8–3.0). Moreover, the observed alternation of the dominant strain among epidemics emerges naturally from the best fit model. Alternative modelling that also includes seasonal forcing in transmissibility shows that both external mechanisms (i.e. seasonal forcing) and the intrinsic mechanisms (i.e., strain interactions) are equally able to generate the observed time-series in seasonal flu.

**Conclusions:**

The intrinsic mechanism of strain interactions alone can generate the observed patterns of seasonal flu epidemics, but according to Kullback-Leibler information distance the importance of extrinsic mechanisms cannot be excluded. The intrinsic mechanism illustrated here to explain seasonal flu may also apply to other infectious diseases caused by polymorphic pathogens.

## Introduction

The incidence of many infectious diseases varies periodically: for example, seasonal influenza develops as an epidemic during winter in temperate regions but remains at very low levels during summer. Furthermore, in infectious diseases caused by multi-strain pathogens such as viral aseptic meningitis [1,2], respiratory syncytial virus [3], cholera [4], influenza [5,6], dengue [7] and rotavirus [8], the dominant strain can also alternate between epidemics although the frequency of alternation is lower than the frequency of epidemics. For convenience, in this study we define ‘epidemic cycling’ as a combination of both periodicities in incidence and alternation of the dominant strain to reflect that successive epidemics is often accompanied by dominant strain alternation (cf. [9]). Despite the common nature of epidemic cycling, their underlying mechanisms are not well understood. Several external mechanisms have been suggested by others for periodicity in infection incidence: e.g., survival of disease pathogen outside host; host behaviour and seasonal changes in host susceptibility and immune defence [10,11,12]. These mechanisms can be expressed as seasonal dynamics [13] caused by the ‘seasonal forcing’ in the transmission rate [3,14]. However, they have difficulty in explaining, for example, a fast and wide spread of influenza [15]. Many human infectious diseases are caused by pathogens that have multiple strains that differ antigenically. And coinfection with different strains is also a common occurrence. This suggests that interactions between strains might play some role in the formation of seasonality [16]. In fact, Grassly et al. [17] showed that it is the intrinsic factors (e.g., immunity), rather than external factors (e.g., changes in human sexual behaviours), that causes an 8–11 year cycle in syphilis incidence. Interactions among different types and subtypes of influenza virus surely play some role in determining the cyclical pattern of incidence and the replacement of the dominant strain [18]. For example, A/H1N1 pdm09 strain emerged in 2009 to cause the 2009 pandemic influenza and displaced the A/H1N1/77 strain which circulated before 2009; while A/H3N2 still circulated [19]. These phenomena could not be due to external factors such as environmental variations and host behaviour changes alone. Therefore traditional seasonal dynamics cannot explain what we call epidemic cycling. Others have suggested that strain interactions such as cross-protective immunity may be responsible for the replacement and cycle of strains (cf. [3,14]). In this study, we consider seasonal influenza as an example and show how strain interactions alone can generate the observed patterns of total and strain-specific incidence of seasonal influenza epidemics.

Seasonal influenza causes approximately 250,000–500,000 deaths globally each year [20]. The mechanisms underlying the annual behaviour of influenza infection are crucial for forecasting and planning for seasonal influenza epidemics. Seasonal influenza epidemics are usually caused by influenza B virus and/or one of two influenza A subtypes: A/H3N2 and A/H1N1. Influenza viruses A and B are very similar in overall structure with eight single-stranded negative-sense RNA segments and only matrix genes differing somewhat between them. The targets of antibodies are two major surface glycoproteins: hemagglutinin (HA) and neuraminidase (NA), which allows for the similarity of possible immunity between types and that between subtypes of type A [21, 22, 23]. For simplicity, type or subtype is in this article roughly referred to as "strain" in view of their similar dynamical behaviours although their biological details are different (c.f. [18, 21, 24]). Detailed studies illustrate that the dominant strain in seasonal influenza cycles irregularly among years [5, 6, 18, 19, 25]. Strains interact by the means of changing human susceptibility and strain infectivity. For example, cross-immunity induced by previous infection protects against subsequent infection [22, 26, 27, 28, 29, 23, 30, 31]. This protection is expressed as either reduced susceptibility [32] or attenuated symptoms during subsequent infections [33].

Nevertheless, infectivity enhancement was observed following a deliberate infection of another strain to previously infected animals (e.g., [34, 35, 36, 37]). A vaccine effectiveness analysis indicates that vaccination can increase the susceptibility to other strains if the vaccine strain mismatches the circulated strains, which occurs in 26% of their study period of 33 years [38, 39]. Increasing evidence emerges to support these observations: Skowronski et al. [40] and Tsuchihashi et al [41] showed that seasonal influenza vaccination might increase susceptibility to A(H1N1) pdm09. Skowronski et al (2013) [42] pointed out that H7N9 infection age profile in China 2013 that was skewed to the older side might hint the phenomenon of cross-reacting antibodies that facilitate infections. Furthermore, Dutry et al. [43] demonstrated that prior addition of human serum to the inoculum trigged a 2–5 fold increase in infected cells. This is thought to occur when low-levels of different but similar antibodies are cross-reactive but not cross protective. When antibodies generated by past exposure to virus antigen form bridging complexes they facilitate uptake and replication of related but non-identical variants of themselves [40].

Coinfection (by which we mean that individuals are simultaneously infected with different strains), hereafter defined as ‘concurrent infection’, was observed in influenza (e.g., [32, 33, 44, 45, 46, 47, 48, 49, 50, 51, 52, 53, 54, 55, 56, 57, 58, 59]). Concurrent infection can be produced by either the simultaneous transmission of two strains [60] or two separate transmissions with the transmission of the second strain before recovery from the first strain. This is different to secondary infection after individuals have recovered from primary infection which is referred to as sequential infection (or re-infection). Within concurrent infections, strains may interact with each other in a way different from how they interact when infections are sequential. This can be argued from the following points: first, as Liu et al. [60] noticed, within two days of symptom onset, no patients infected with influenza A virus had detectable hemagglutination inhibition (HI) antibodies against other strains of influenza; second, through better aerosolisation infection with one strain of influenza may increase the chance of being infected with another strain of influenza during the infectious period of the first infection [61]. Though Brundage [61] focused on the interactions between influenza viruses and bacterial respiratory pathogens, the same argument might also apply to the interaction between influenza viruses through the better aerosolisation. These two points might imply the possibility of infectivity enhancement within concurrent infection. So far there is no direct report showing infectivity enhancement within concurrent infection in influenza, which is perhaps because of the short duration of influenza virus infectiousness (typically less than a week). Based on the observed data [60] of concurrent infection and co-transmission events, Zhang and De Angelis [62] demonstrate an indirect evidence of infectivity enhancement during concurrent infection in flu. As a theoretical exploration, we will in this paper investigate how this possible interaction along with the well-known cross-immunity affects dynamic patterns of season flu.

Cross-immunity during sequential infection has been widely recognised in multi-strain transmission models (e.g., [14, 63, 64]) while the possible infectivity enhancement within concurrent infection has only recently been noticed [16]. These strain interactions might collectively provide an explanation for the periodicity in incidence and alternation of the dominant strain in seasonal influenza. Truscott et al. [14] proposed a two strain SIRS epidemic model that allows for age and includes cross-immunity and seasonal forcing. They can almost generate the observed patterns of seasonal influenza: dominant strain alternation in successive years is due to a negative association between strains which is generated by cross-immunity; and incidence periodicity is due to seasonal forcing and variation between age groups of the contact rate, infectivity and susceptibility. However, the reports of seasonal influenza usually include three strains (i.e. type B, A/H1N1 and A/H3N2) and the alternation of dominant strains is not as regular as Truscott et al. [14] predicted (i.e., [5, 6, 18, 25]).

In this study seasonal influenza epidemics are modelled via a three strain SIRS model. It is worth mentioning that small changes in the three routinely reported strains occur continuously due to frequent antigenic drift caused by mutation in the viral genome. Immunity built through primary infection will wane either because of immune loss within the human body or immune escapement due to changes in the strains. To approximate these complications, the model assumes a constant biological identity for each strain but allows for a waning immunity. Novel influenza virus strains can also be generated due to antigenic shift via reassortment, which could cause pandemic influenza. Because antigenic shift is much rarer than antigenic drift, it is ignored in this study. The emergence of pandemic influenza due to reassortment was discussed in a previous study [65].

Within this model two types of strain interaction are assumed: (a) the immunity due to a past infection of a different strain (referred to as “cross-immunity”); and (b) the greater infectivity of an individual who is simultaneously infected with more than one strain (referred to here as “enhanced infectivity within concurrent infection”) (cf. [16]). Strain interaction within concurrent infection can induce the periodic epidemics; however, strains become synchronized if cross-immunity is sufficiently strong (cf. [16]). Like Truscott et al. [14], we assumed that seasonal influenza was typically characterised by an outbreak duration of 11 weeks and an annual attack rate of 15% and an epidemic period of one year. The possible range for model parameters was determined by using the Kullback-Leibler information distance, which is based on a comparison between the predicted epidemics and the observed epidemics of seasonal influenza. In addition the replacement of the dominant strain was used as another criterion for the goodness of fit. The model we propose here includes two different types of parameter. One type is used to describe the traditional transmission characteristics such as the transmission rate, the infectious period and the immunity period; the second type is used to represent strain interactions such as co-transmissibility, infectivity enhancement within concurrent infection and cross-immunity during sequential infection. The purpose of the model fitting exercise is to show whether the model proposed here can explain the typical patterns of seasonal influenza under the widely accepted values of transmission characteristics and reasonable ranges of strain interaction parameters.

## Models and Methods

**Fig 1:**
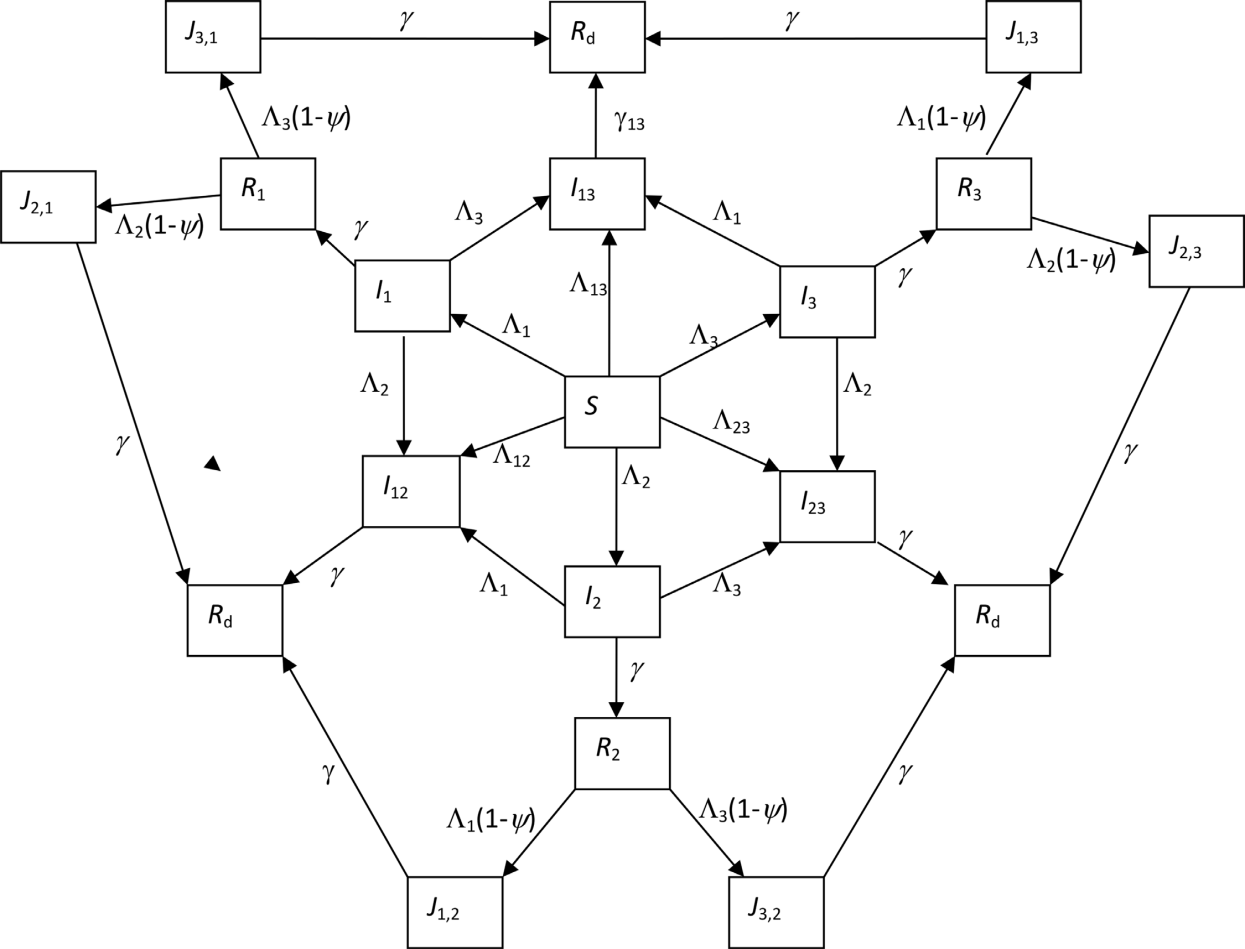
Flow chart of the three-strain SIRS epidemic model. Solid arrows indicate transitions. Expressions next to arrows show the *per capita* flow rate between compartments. Births and deaths, and transitions from the recovered to the susceptible are not shown. Variables and parameters are explained in Tables 1 and 2. Triple infection is ignored by assuming that, once infected with two strains (either concurrent or sequential), no one can be infected by the third strain.

The flowchart of the three strain SIRS model is shown in Fig 1 where two types of strain interaction are assumed: cross-immunity and infectivity enhancement (cf. [16]). The model has 17 compartments as shown in Table 1 and the model parameters are as defined in Table 2. For simplicity, the three strains are assumed to be phenotypically indistinguishable. Some experimental evidence exists that cross-immunity will increase with repeated infections (e.g., [21, 22, 23]). We hence assume that once infected with two strains, individuals cannot be further infected by the third strain. That is, those who have recovered from single infections become fully immune to the infecting strain and partially immune to other strains with reduction *ψ* in susceptibility during the immunity period *D*; while those who have recovered from dual infections become completely immune to all the three strains. The immunity within compartments *R*_*i*_, *i* = 1, 2, 3, and *R*_d_ are assumed to wane at the same rate *σ* and all different infections are assumed to have the same infectious period *d*_I_. The infectivity enhancement of strain *i* within concurrent infection *I*_*ij*_ is measured by a coefficient *ϕ*. Assuming symmetry among the three strains, the increased infectivity is the same to strain *i* and *j* within a concurrent infection *I*_*ij*_. The additional mortality caused by the virulence of infections is ignored, and births and deaths are assumed to be balanced in order to maintain a constant population size. Age and spatial heterogeneity are ignored so that homogeneous mixing of the population is assumed. To avoid the “trough extinction” an external force of infection (EFOI) (*ε*) representing the effect of contact between the modelled population and the infected from outside is included but is assumed to cause only single-infected patients (cf. [14]). The model system is described by the following set of differential equations:

$$\frac{dS}{dt}=(1-S)/L-(\sum_{i}\Lambda_{i}+\sum_{i<j}\Lambda_{ij}+3\varepsilon )S+(\sum_{i}R_{i}+R_{\text{d}})/D$$

$$\frac{dI_{i}}{dt}=(\Lambda_{i}+\varepsilon )S-(\sum_{j\neq i}\Lambda_{j})I_{i}-(1/d_{\text{I}}+1/L)I_{i},i=1,2,3$$

$$\frac{dI_{ij}}{dt}=\Lambda_{j}I_{i}+\Lambda_{i}I_{j}+\Lambda_{ij}S-(1/d_{\text{I}}+1/L)I_{ij},i<j$$

$$\frac{dJ_{i,j}}{dt}=(1-\psi )[\Lambda_{i}+\varepsilon ]R_{j}-(1/d_{\text{I}}+1/L)J_{i,j},i\neq j$$ (1)

$$\frac{dR_{i}}{dt}=\gamma I_{i}-[(1-\psi )(\sum_{j\neq i}\Lambda_{j}+2\varepsilon )+1/D+1/L]R_{i}$$

$$\frac{dR_{\text{d}}}{dt}=\gamma (\sum_{j\neq i}J_{i,j}+\sum_{i<j}I_{ij})-(1/D+1/L)R_{\text{d}}$$

Where

$$\Lambda_{i}=\beta (I_{i}+\sum_{j\neq i}J_{i,j}+\varphi \sum_{j_{1}<j_{2};j_{1},or,j_{2}=i}I_{j_{1}j_{2}}),i=1,2,3$$ (2)

are the force of infection of the three single strains,

$$\Lambda_{ij}=\beta_{\text{d}}I_{ij},i<j$$ (3)

are the force of infection of the three dual infections.

**Table 1:** Model Variables.

Variable	Definition
*S*	Proportion of the population that are susceptible to all strains
*I*_*i*_	Proportion of primary infections with strain *i* = {1,2,3}
*I*_*ij*_	Proportion of concurrent infections with two strains *i* and *j* (>*i*)
*J*_i,j_	Proportion of secondary infections with strain *i* after recovery from infection with strain *j*.
*R*_*i*_	Proportion of those recovered from single infections with strain *i* so that they are immune to strain *i*
*R*_d_	Proportion of these recovered from double infections and now are immune against any further infection

**Table 2:** Model parameters and their baseline values. The values shown are those used if not otherwise specified. The baseline values are extracted from Truscott et al. [14] and Boëlle et al [66] except *β*_d_ and *ϕ* whose baseline values are tentatively chosen.

Parameter	Definition	Baseline value
*β*	Transmission rate for single strain	1.0 per person per day
*β*_d_	Co-transmission rate of double infection	0.25 per person per day
*ϕ*	Infectivity enhancement of single strain within concurrent infection	2.5
*ψ*	Cross-immunity induced by primary infection	0.7
*D*	Immunity duration (1/*σ*) with *σ* representing the waning rate of immunity	5 years
*d*_I_	Infectious period (1/*γ*) with *γ* representing the recovery rate	2 days
*L*	Life span (1*/μ*) with *μ* representing the birth rate	70 years
*N*	Population size	6.3×10^7^
*δ*	Relative amplitude of variation in the transmission rate	0
*ε*	External force of infection	4.1×10^−9^ per person per day

In an additional model, we also examine the effect of seasonal forcing in the transmission rate on the patterns of seasonal influenza by considering

β(t)=β(1+δcos(2πt/365)) (4)

Here *δ* is the relative amplitude, which reflects the annual variation in contact intensity and environmental conditions, and *β* is the average value of the transmission rate. Another similar time-varying contact rate is also included for the co-transmission rate *β*_d_.

Strain interactions are characterised by three parameters: co-transmission rate (*β*_d_), coefficient of infectivity enhancement within concurrent infection (*ϕ*) and cross-immunity during sequential infection (*ψ*). It is worth mentioning that because the infectivity enhancement *ϕ* only directly benefits the transmission of single strains the relative prevalence of concurrent infection (*I*_*ij*_) is mainly determined by *β*_d_ rather than *ϕ* as illustrated in Table 2 of [16]. Therefore the low number of reported concurrent infections (e.g., [32, 60]) should not simply be regarded as a fact for rejecting the infectivity enhancement of single strains which we assumed in this study.

To generate a long-term dynamic process of infection, infection was initiated by randomly selected seed infections of each type and a burn-in period of 20000 years is allowed to let the system be fully developed. The time series of infection was monitored to detect the times at which the incidence rate rose above or fell below a threshold level of 42 cases per 100000 person-days (cf. [14]) so to obtain the duration of epidemic (DE) and the inter-epidemic period (IEP). The attack rate (AR) is defined as the proportion of the population infected during the epidemic. These three characteristics were calculated only for the total number of all infections. Stochastic extinction occurs once the total number of infected people reduces to below one. Following Truscott et al. [14], the Kullback-Leibler (KL) information distance over parameter space was calculated to measure the match between the model outputs and these three characteristic features,

$$\text{KL}(g(\pi ),f)=\int g(\pi )\;\text{log}(\frac{g(\pi )}{f})dx$$ (5)

where *f* is the empirical distribution of a feature, and g(*π*) is the approximate distribution of the same feature from model simulations over 1000 consecutive epidemics under the values of a set of model parameters *π*. As in Truscott et al. [14], empirical patterns of seasonal influenza are assumed to be characterised by these three normally distributed features with mean (*m*) and standard deviation (SD): (*m*, SD) = (11,3) weeks for DE, (15%,5%) for AR, and (52,7) weeks for IEP. We approximate the continuous empirical distribution *f* as *f*_*i*_, *i* = 1,…,*N*_bin_ where *N*_bin_ is the number of bins. Under the values of the model parameters *π*, the predicted distribution is categorised as *g*_*i*_ where *i* = 1,…,*N*_bin_. The KL information distance is approximated as

$$\text{KL}(g(\pi ),f)\approx \sum_{i=1}^{N_{\text{bin}}}g_{i}\;\text{log}(g_{i}/f_{i})$$ (6)

*N*_bin_ takes the following values: 52, 104 and 100 for DE, IEP and AR, respectively. The overall measure of goodness of fit is an unweighted sum of the information distance for DE, AR, and IEP, denoted in this paper as KL3. For convenience, acronyms that are used to describe the characteristic features of an infection time series (such as DE) are listed in Table 3.

**Table 3:** Acronyms used for characteristic features of epidemic time series.

Acronym	Definition
DE	Duration of an epidemic defined as the time interval when the daily number of infections continuously exceeds an epidemic threshold level of 42 per 100000 persons per day.
KL*_*DE	Kullback-Leibler (KL) information distance from a comparison of the observed DE and the predicted DE.
AR	Attack rate defined as the proportion of the population infected during an epidemic
KL*_*AR	KL information distance from a comparison of the observed AR and the predicted AR.
IEP	Inter-epidemic period
KL_IEP	KL information distance from a comparison of the observed IEP and the predicted IEP
KL3	Combined KL information distance defined as an unweighted sum of the three KL component information distances: KL_DE+KL_AR+KL_IEP

The baseline values of model parameters given in Table 2 are based on recent reviews of influenza transmission parameters [14, 66]. Derived from the baseline values is the basic reproductive number of single strains, defined as the average number of secondary infections caused by an infected patient within a naïve population, *R*_0_ = *β*/(*γ*+*μ*) = 2.0. We assume a baseline value of 0.25*β* for co-transmission rate *β*_d_ so the corresponding reproductive number of dual infection, $R_{0}^{\text{d}}=\beta_{\text{d}}/(\gamma +\mu )$ = 0.5. Further the baseline value for infectivity enhancement *ϕ* is tentatively assumed to be 2.5, which is confirmed by simulations shown below to be within the range of values that can generate practical patterns of seasonal influenza. Simulations were run within a population of the size of the UK (*N* = 63 million). We investigated parameter space especially to see which values of the strain interaction parameters (*β*_d_, *ϕ*, and *ψ*) generated cyclical or chaotic epidemics [14, 16], which most closely resembled empirical patterns. The examples of how infectivity enhancement and cross-immunity interact to produce different types of epidemic are given in S1 File.

Under the circumstance of no seasonal forcing (i.e., *δ* = 0) and weak infectivity enhancement (e.g., *ϕ* <1.5 in S1 Fig), only endemics with constant incidence where the number of new infections is balanced by the number of recoveries are possible. When the infectivity enhancement exceeds a certain threshold *ϕ*_c_ (i.e., 1.7 by assuming the baseline values for other model parameters) the endemics bifurcate into cyclical (recurrent) epidemics. However, as *ϕ* further increases, the cyclical epidemics burst into chaotic epidemics (S1 Fig and S2A Fig). When the cross-immunity is very strong (i.e., *ψ* >80%) only cyclical epidemics are possible as shown in S1 Fig. With an increased co-transmissibility (*β*_d_), the threshold infectivity enhancement *ϕ*_c_ reduces (cf. Fig 4A of [16]). This implies that both infectivity enhancement and co-transmission complement each other to sustain cyclical epidemics. We notice that when the periodic epidemics are regular, the goodness of fit is not as good as for chaotic epidemics (see S2E Fig). Clearly the three characteristics of the seasonal flu patterns (i.e., DE, AR and IEP) vary between epidemics when they are chaotic or aperiodic (see S2B–S2D Fig) as observed in influenza surveillance [5, 6, 19, 25]. In the following we judge the model output by the combined KL information distance (KL3).

We define a strain as the dominant strain if the fraction of infections with that strain exceeds 50% during an epidemic. Under the situation of synchronous strains, the three strains have the same share of the total infection at any time. With asynchronised strains the fraction of infections of each strain varies between epidemics. This fraction does not necessarily exceed 50% and therefore there is not necessarily any dominant strain. It is obvious that within the three strain system, the requirement for the alternation of dominant strains is stronger than the emergence of asynchronous strains, which is clearly illustrated in S1 and S3 Figs.

## Results

**Fig 2:**
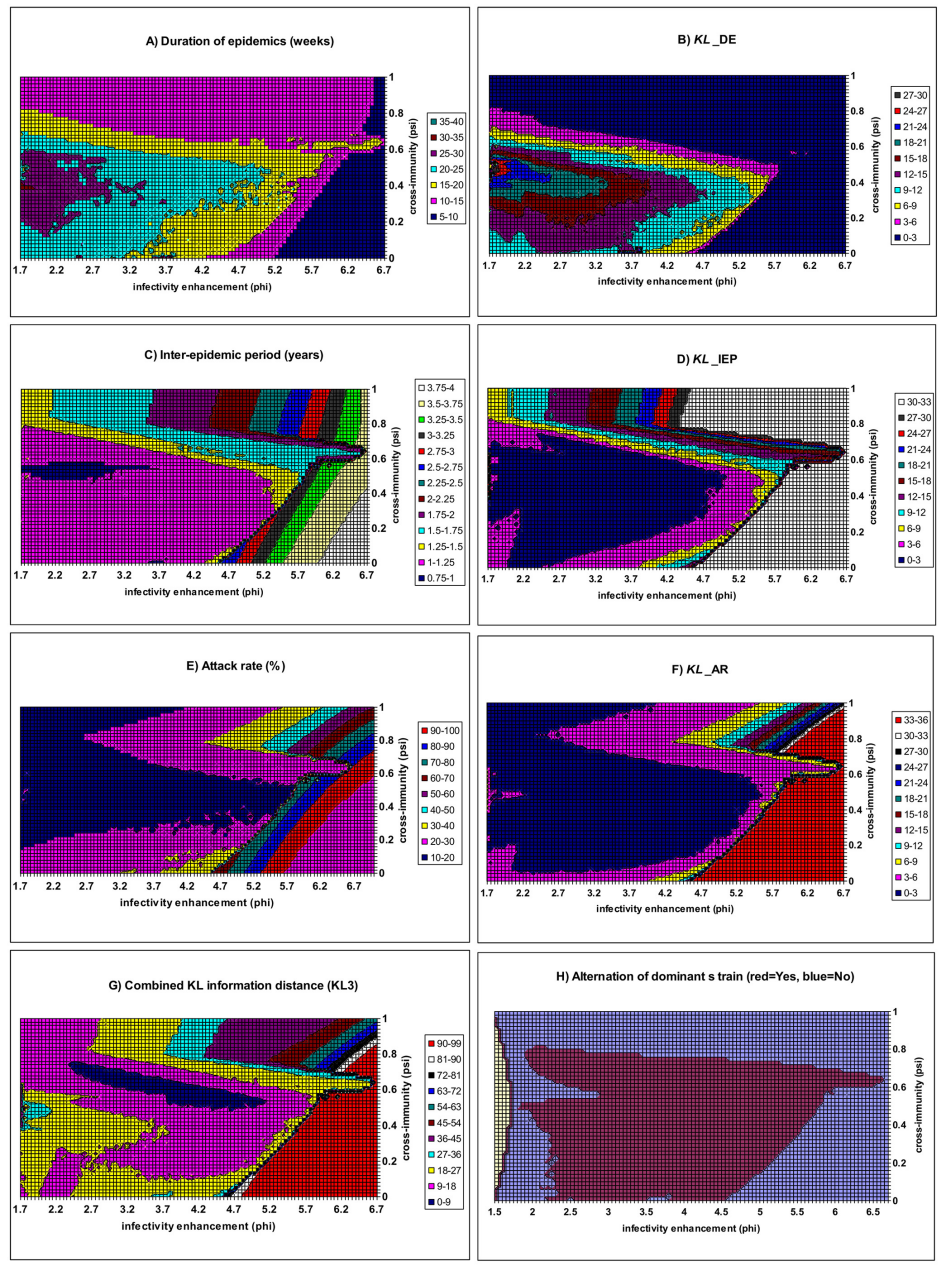
Model fit as a function of cross-immunity and infectivity enhancement. Other parameters are as baseline values in Table 2. A) Duration of epidemics (DE) in weeks; B) Goodness of fit as measured by the KL information distance of duration of epidemics (KL_DE); C) Inter-epidemic period in years; D) Goodness of fit as measured by the KL information distance of inter-epidemic period (KL_IEP); E) Attack rate; F) Goodness of fit as measured by the KL information distance of attack rate (KL_AR); G) the overall goodness of fit to the three characteristics (KL3); H) alternation of the dominant strain (the yellow area represents the model parameter region where only endemics with constant incidence are available).

Fig 2 shows the exploration of parameter values for cross-immunity (*ψ*) and infectivity enhancement (*ϕ*). Over a wide range of values for *ψ* and *ϕ*, the distribution of the KL information distance for each of the three characteristic features was plotted. On the upper and right side of Fig 2B is the area for which KL_DE is less than 3.0. On the corresponding area of Fig 2A, the mean duration of the epidemics (DE) ranges from 5 to 20 weeks and matches the observed values. On the bottom left corner of Fig 2D and 2C where KL_IEP < 3.0, the mean inter-epidemic period (IEP) ranges from 1.0 to 1.25 years. On the top left corner of Fig 2F and 2E on which KL_AR < 3.0, the attack rate (AR) ranges from 10 to 20%. The overall measure of goodness of fit (KL3) is plotted on Fig 2G. The best fit region is located within a narrow band: 0.5<*ψ*<0.75 and 2.4<*ϕ* <5.0 on which KL3 is less than 9.0. Within this narrow band, the duration of epidemics (DE) is estimated to range from 15 to 20 weeks; the inter-epidemic period (IEP) ranges from 1.0 to 1.3 years and the attack rate (AR) ranges from 15% to 25%. IEP estimates appear longer than the observed, however, the overall model outputs are a reasonable match to the empirical patterns. Model outputs are much less likely to match the empirical patterns for parameter values on which KL3 > 9.0 (Fig 2). Fig 2H shows that when cross-immunity is very strong (i.e. >80%), or infectivity enhancement (*ϕ*) is not strong (i.e., <1.7), the dominant strain cannot change (cf. [16, 67]). While for a very weak cross-immunity, the dominant strain changes between epidemics but KL3 is unacceptably high, implying that the model outputs deviate substantially away from the typical observations of seasonal flu.

**Fig 3:**
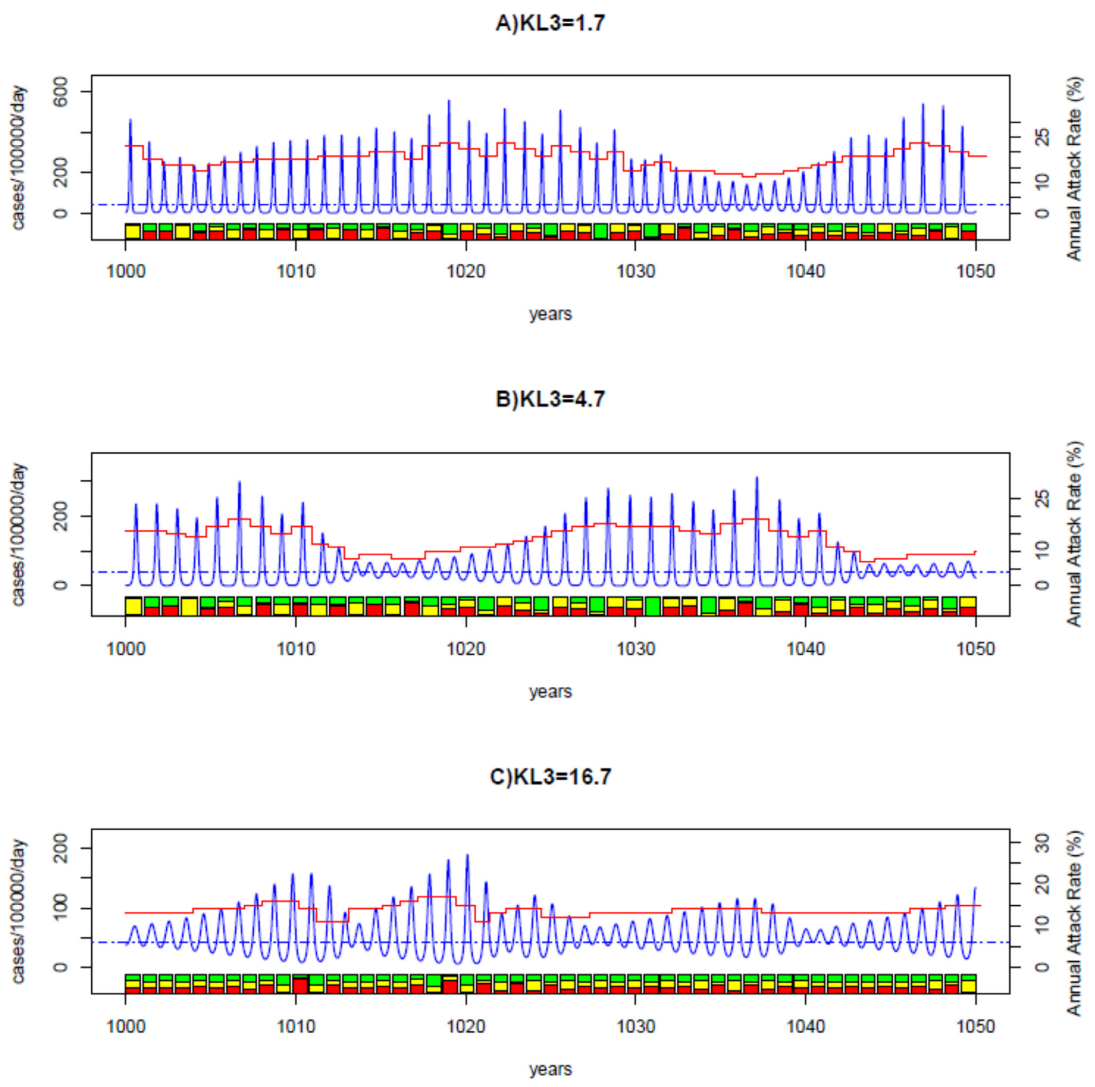
Examples of model output time series. The blue lines show the total case incidence, the red lines the annual attack rate and the bottom bar plot shows the fractions of the three strains (represented by three different colours). The dashed blue straight line represents the threshold level of 42 cases per 100000 person-days for defining an epidemic. Other parameters are as baseline values in Table 2. Model parameters: A) *R*_0_ = 2.5, *D* = 4.5 years, *ψ* = 0.75, *ϕ* = 2.3. The infection time series generated has a mean IEP of 1.0 year, a mean DE of 14 weeks and a mean AR of 18%, with its overall goodness of fit: KL3 = 1.7 (KL_DE = 1.1, KL_IEP = 0.2, KL_AR = 0.4). B) *R*_0_ = 2.0, *D* = 5.0 years, *ψ* = 0.70, *ϕ* = 2.5. The infection time series generated has a mean IEP of 1.1 years, a mean DE of 19 weeks and a mean AR of 13%, with its KL3 = 4.7 (KL_DE = 3.1, KL_IEP = 0.7, KL_AR = 0.2); C) *R*_0_ = 2.0, *D* = 5.0 years, *ψ* = 0.50, *ϕ* = 2.5. The infection time series generated has a mean IEP of 1.0 year, a mean DE of 27 weeks and a mean AR of 13%, with its KL3 = 16.7 (KL_DE = 14.7, KL_IEP = 0.7, KL_AR = 1.2).

Three examples of model output time series are shown in Fig 3. When KL3 is small, the three characteristic features produced by the model match well those of empirical patterns. For the example shown in Fig 3A in which KL3 = 1.7, the output time series has a mean IEP of 51 weeks and a mean DE of 14 weeks, and a mean AR of 18%. Furthermore, the dominant strain changes irregularly between epidemics. A time series with KL3 <9.0 can still resemble the empirical patterns. For the example in Fig 3B where KL3 = 4.7: the average IEP was 57 weeks and the average DE was 19 weeks which are both slightly longer than observed. When KL3 >9.0 the model outputs deviate from the observed patterns. For the example in Fig 3C, the epidemics have a mean IEP of 52 weeks but have a mean DE of 27 weeks, which is unlikely to be true. We therefore use a tentative criterion of KL3 <9.0 to classify the overall goodness of fit: the output infection time series of KL3 <9.0 corresponding to realistic behaviours with appropriate distributions of the three characteristic features.

**Fig 4:**
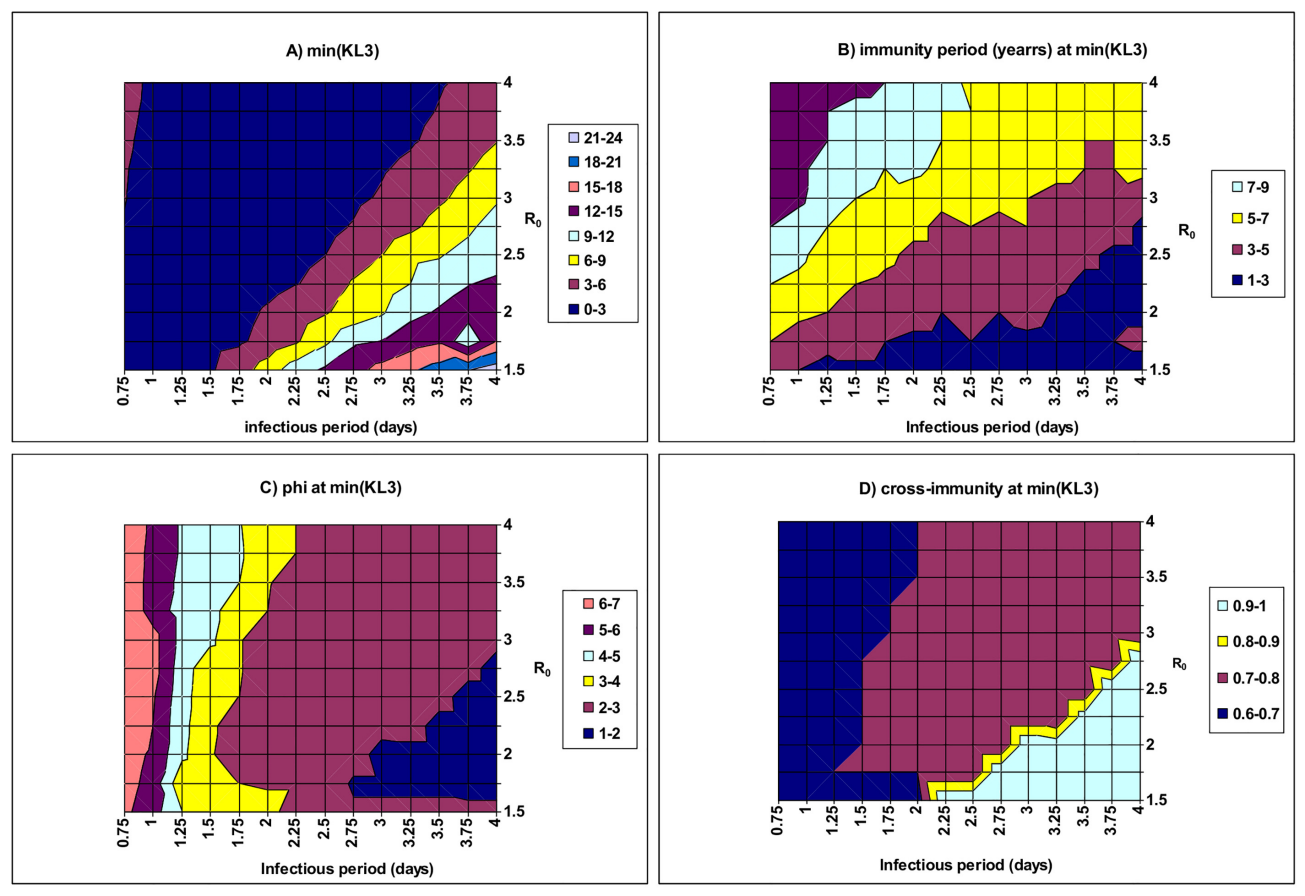
The minima of the combined KL information distance (KL3) as a function of the infectious period (*d*_I_) and the basic reproductive number (*R*_0_). For each pair of *d*_I_ and *R*_0_, the best fit parameters were searched over the following space, *ϕ*: [1,7] gridded evenly into 71 points, *ψ*: [0,1] gridded evenly into 21 points and *D*: [1,10] years gridded evenly into 21 points. It is assumed that the co-transmission rate *β*_d_ = *β*/4 and the external force of infection (*ε*) is fixed at 4.1×10^−9^ per person per day. A) shows that there is a positive correlation between *d*_I_ and *R*_0_: when *R*_0_ is about 1.5, *d*_I_ is <2.1 day; whilst when *R*_0_ = 2.5 *d*_I_ should be <3.5 days. B) shows that the immunity period decreases with *d*_I_ but increases with *R*_0_; C) shows that for a given *R*_0_, infectivity enhancement (*ϕ*) required to generate the observed patterns of seasonal influenza decreases with *d*_I_; D) shows that cross-immunity increases with *d*_I_ but decreases with *R*_0_.

In order to identify the best fitting regions of model parameters, the 5-dimensional parameter space of: infectious period (*d*_I_), basic reproduction number (*R*_0_), infectivity enhancement (*ϕ*), cross-immunity (*ψ*) and immunity period (D) was divided to explore. Fig 4A shows the minima of KL3 and Fig 4B, 4C and 4D show their corresponding best fitting values of the five parameters. A linear relationship emerges between the best fitting values of *R*_0_ and those of *d*_I_ (Fig 4A), which indicates a constant transmission rate *β* because of the formula *R*_0_ = *βd*_I_. The results show that when *R*_0_ is within the accepted range 1.5–3.0 [66], the infectious period is more likely to be shorter than 4 days. It is also found that there is a positive association between the best fitting values of *R*_0_ and those of the immunity period (*D*) (Fig 4B). A higher transmissibility (*R*_0_) is compensated for by a longer immunity period which reduces the susceptible proportion of the population, leading to a constant mean attack rate. Fig 4B also shows that the immunity period (*D*) decreases with the infectious period, which is a consequence of both the constancy of the transmission rate (Fig 4A) and the positive association between *D* and *R*_0_. Infectivity enhancement is negatively associated with the infectious period but cross-immunity is positively associated with the infectious period; however, they both appear to be relatively not associated with *R*_0_ (Fig 4C and 4D). It is worth noting that within the best fitting region (i.e. that of KL3 <9.0 in Fig 4A), infectivity enhancement *ϕ* ranges from 2 to 7 (Fig 4C), the cross-immunity stays within a narrow range: 0.60–0.80 (Fig 4D) with its average duration (*D*) ranging from 3 to 9 years (Fig 4B). Within these regions, model outputs have a mean DE of 8–18 weeks, a mean IEP of 0.9–1.2 years, and a mean AR of 5–30%, which are fairly close to the empirical patterns of seasonal influenza. Furthermore, the dominant strain among the three strains alternate between epidemics within the best fitting model parameters (data not shown). Within the bottom right corner of Fig 4A where strains are of low *R*_0_ but long *d*_I_, the fit of the model is poor: cross-immunity is very strong and the dominant strain does not change between epidemics (cf. S1 Fig). Fixing at other alternative values of both co-transmission rate (*β*_d_) and external force of infection (*ε*), similar explorations in the 5-dimension parameter space suggest the above observations still hold over a wide region of model parameters *β*_d_ and *ε* (see S2 File).

**Fig 5:**
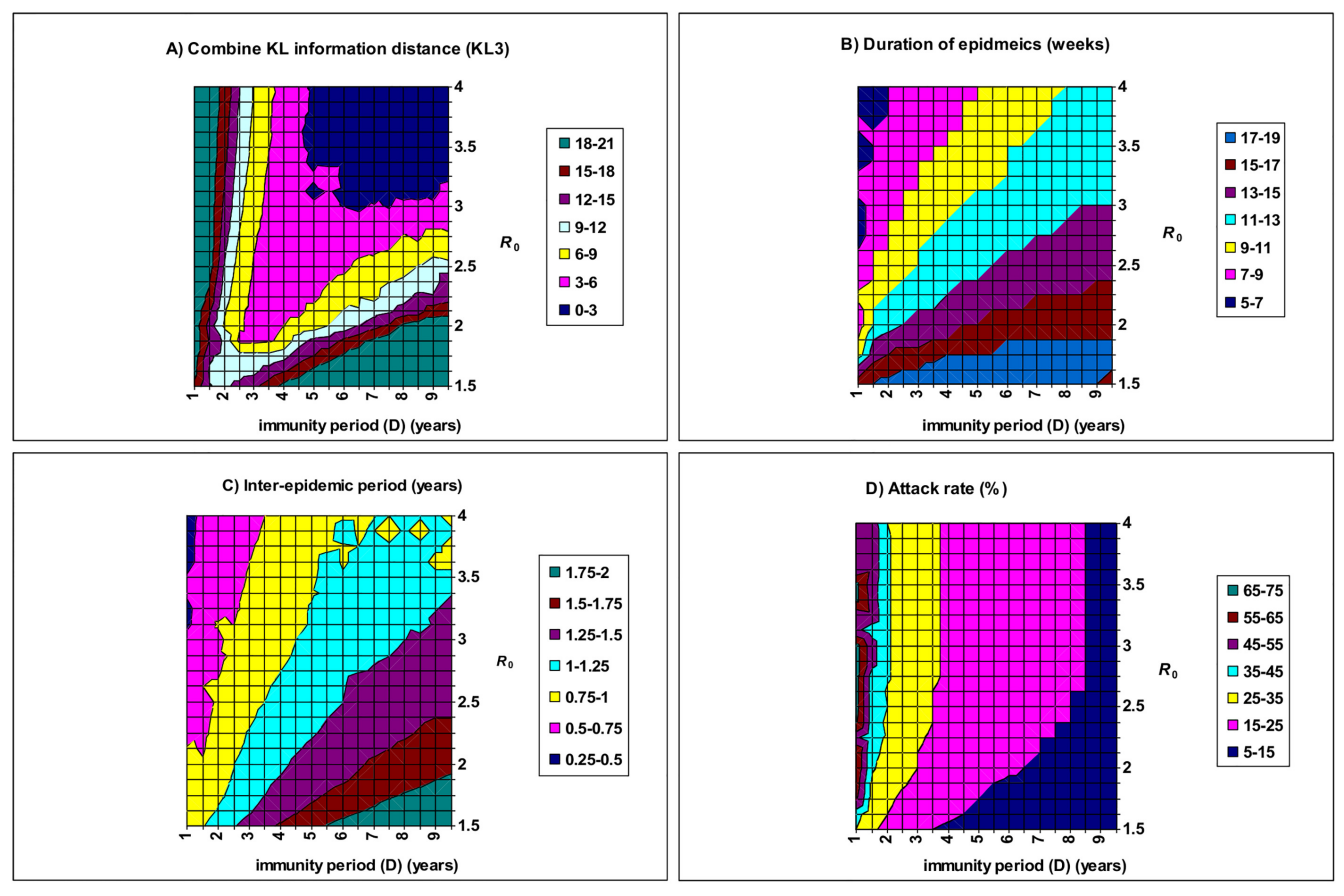
Model fit as a function of the basic reproductive number (*R*_0_) and the immunity period (*D*). It is assumed that the cross-immunity (*ψ*) is fixed at 0.75 and the co-transmission rate (*β*_d_) is equal to *β/*4. Other parameters have the baseline values in Table 2.

Fig 5A illustrates the model fit (KL3) as a function of the basic reproductive number (*R*_0_) and the immunity period (*D*). The best fitting values suggest that a low transmissibility is compensated by a low immunity period (*D*: 2–4 years), while a high transmissibility is associated with a wide range of immunity period (3.0<*D*<10.0 years), as also shown in Fig 4. Both DE and IEP decrease with *R*_0_, but increase with *D* (Fig 5B and 5C). AR is relatively insensitive to *R*_0_ but decreases with *D* (Fig 5D). Fig 5A shows that the model behaves well (with the tentative criterion of KL3 <9.0) in a broad triangular parameter area: 1.8< *R*_0_ <4.0 and 2< *D* < 9 years. Within these parameter areas, DE ranges from 7–15 weeks, IEP from 0.8–1.3 years, and AR from 10–30%. And the dominant strain alternates between the three strains. That is, model epidemics on these parameter regions resemble the observed patterns.

**Fig 6:**
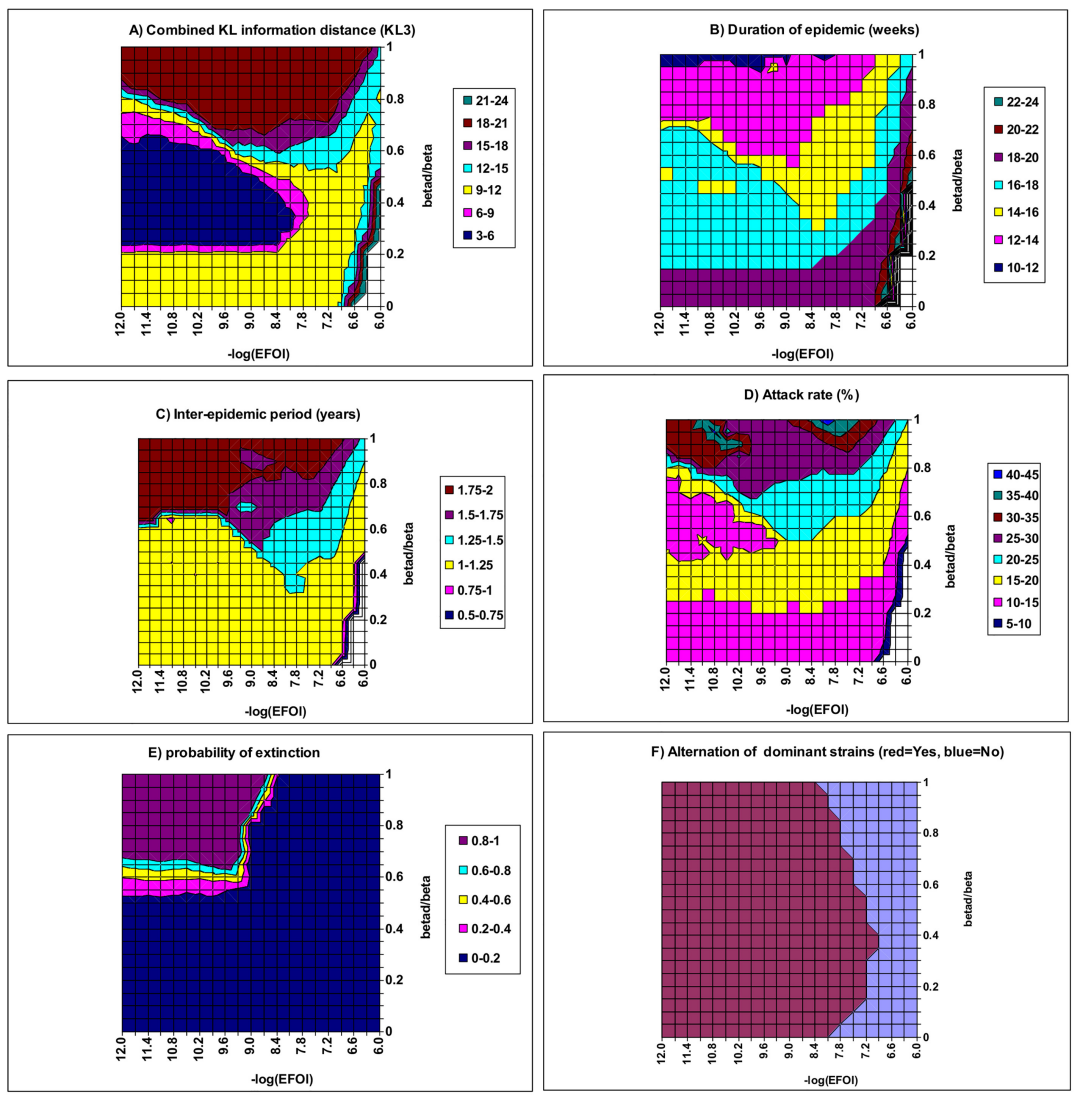
Model fit as a function of the external force of infection (*ε*) and the co-transmission rate (*β*_d_). Other parameters have the baseline values in Table 2. Note that within the small whited triangle of the bottom right corner when the co-transmission rate is weak (*β*_d_ <0.4*β*) but the EFOI is strong (*ε* >10^−7^ per person per day), only endemics with constant incidence where the number of new infections is balanced by the number of recoveries are possible.

Fig 6 explores how the external force of infection (EFOI) and co-transmission rate (*β*_d_) affect the model outputs. When *β*_d_ is weak and EFOI is strong, only endemics with constant incidence are possible (see the small right triangle on the bottom right corner of Fig 6). This indicates that a strong constant EFOI will prevent variations in incidence. It is interesting to note how the effect of *β*_d_ depends on the value of EFOI. When EFOI is larger than 6.3×10^−9^ per day, the three strains remain synchronous and there is no trough extinction. This is because, at these high levels of EFOI, there are more than three cases imported per week. When EFOI is smaller than 6.3×10^−9^, the infection cannot become extinct when *β*_d_ is low; however, as *β*_d_ exceeds ½*β*, the infection becomes increasingly likely to suffer extinction (Fig 6E; [16]). On this small range of EFOI, epidemic patterns remain roughly unchanged because the level of EFOI is negligible compared with the average force of infection generated by the indigenous population. Based on the value of KL3 and the probability of extinction (<10%) (Fig 6A and 6E), the best fitting values of *β*_d_ are 0.2*β*–0.5*β* (i.e., *R*_0_^d^ from 0.4 to 1.0) with EFOI (*ε*) <6.3×10^−9^ per day under the situation of Fig 6. Within this region of model parameter values, model outputs have a mean DE of 10–16 weeks, a mean IEP of 1.0–1.3 years, and a mean AR of 10–25%. These are close to the empirical patterns of seasonal flu. Note that the range of EFOI estimated in this study includes the maximum likelihood estimate obtained by Truscott et al. [14]: 5.5×10^−9^ per day.

**Fig 7:**
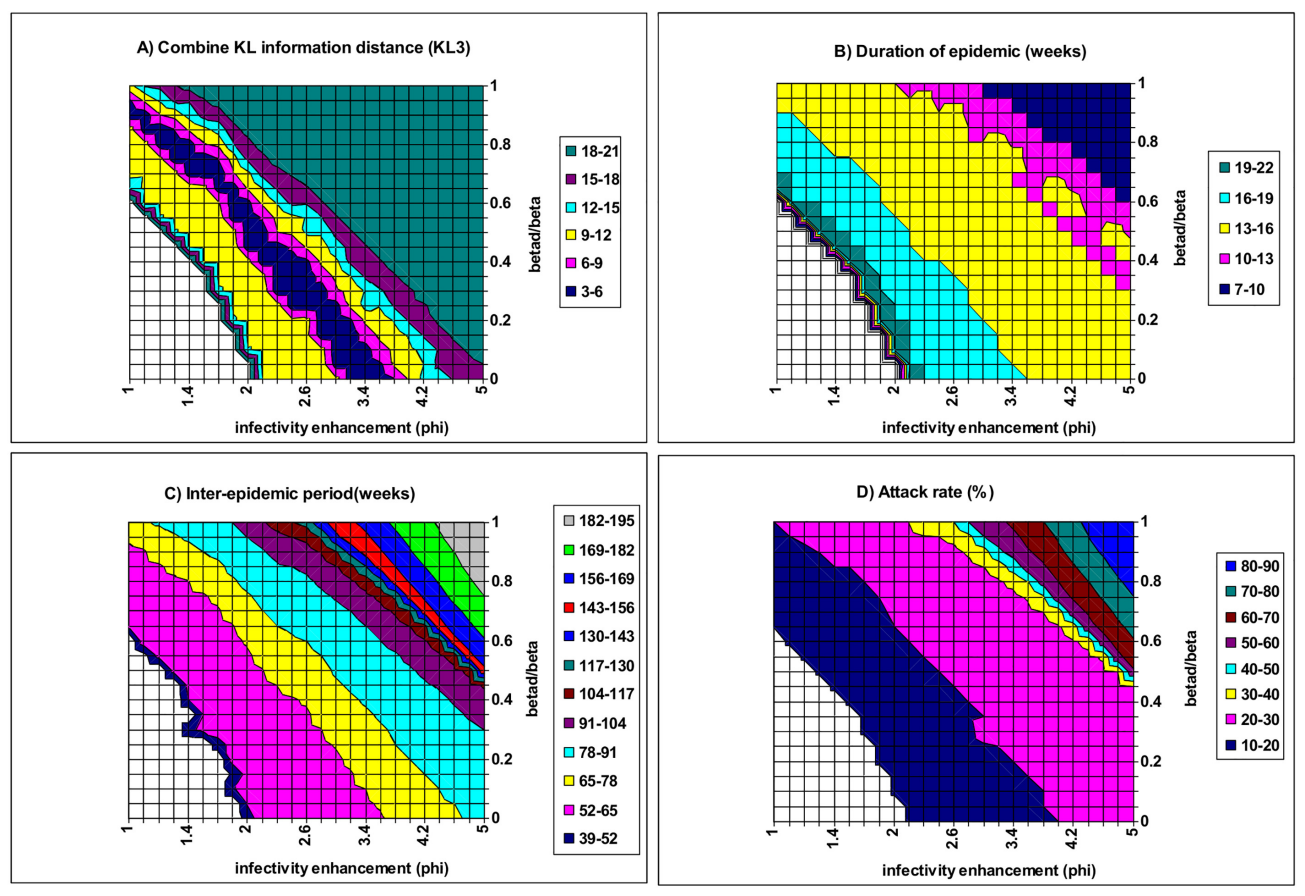
Model fit as a function of the infectivity enhancement (*ϕ*) and the co-transmission rate (*β*_d_) to demonstrate the effect of strain interaction within concurrent infection. Other parameters have the baseline values in Table 2. A) the combined KL information distance (KL3); B) duration of epidemic; C) inter-epidemic period; D) attack rate. Within the whited triangular region of the bottom left corner, only endemics with constant incidence where the number of new infections is balanced by the number of recoveries are possible.

Fig 7 illustrates the effect of strain interactions within concurrent infection on the model outputs. When both *ϕ* and *β*_d_ are weak (i.e., the bottom left corner), cyclical epidemics cannot occur. As *ϕ* and *β*_d_ increase to exceed certain threshold values, cyclical or chaotic epidemics will occur (cf., S1 Fig, and Fig 4 of [16]). When they become very large (i.e., the right top corner), IEP increases to three years or longer (Fig 7C) while the DE shortens to 6–9 weeks (Fig 7B) and AR exceeds 80% (Fig 7D). Under these circumstances, the infection incidence increases to very high levels within a reduced DE while they fall to very low levels during a long inter-epidemic period so that they can easily become extinct (cf. S3H Fig). Between these two extreme regions in the *ϕ*–*β*_d_ plane, model outputs resemble reasonably well the empirical patterns of seasonal flu. Within a narrow diagonal band of KL3 <9.0, model outputs are a good resemblance to the observed patterns with a mean DE of 15–18 weeks, a mean IEP of 1.0–1.5 years and a mean AR of 10–20%. Within this band, there is a negative association between *ϕ* and *β*_d_, implying a compromise between the two aspects of strain interaction within concurrent infection. Further the dominant strain alternates among the three strains between epidemics when both *ϕ* and *β*_d_ take values from the band (data not shown).

**Fig 8:**
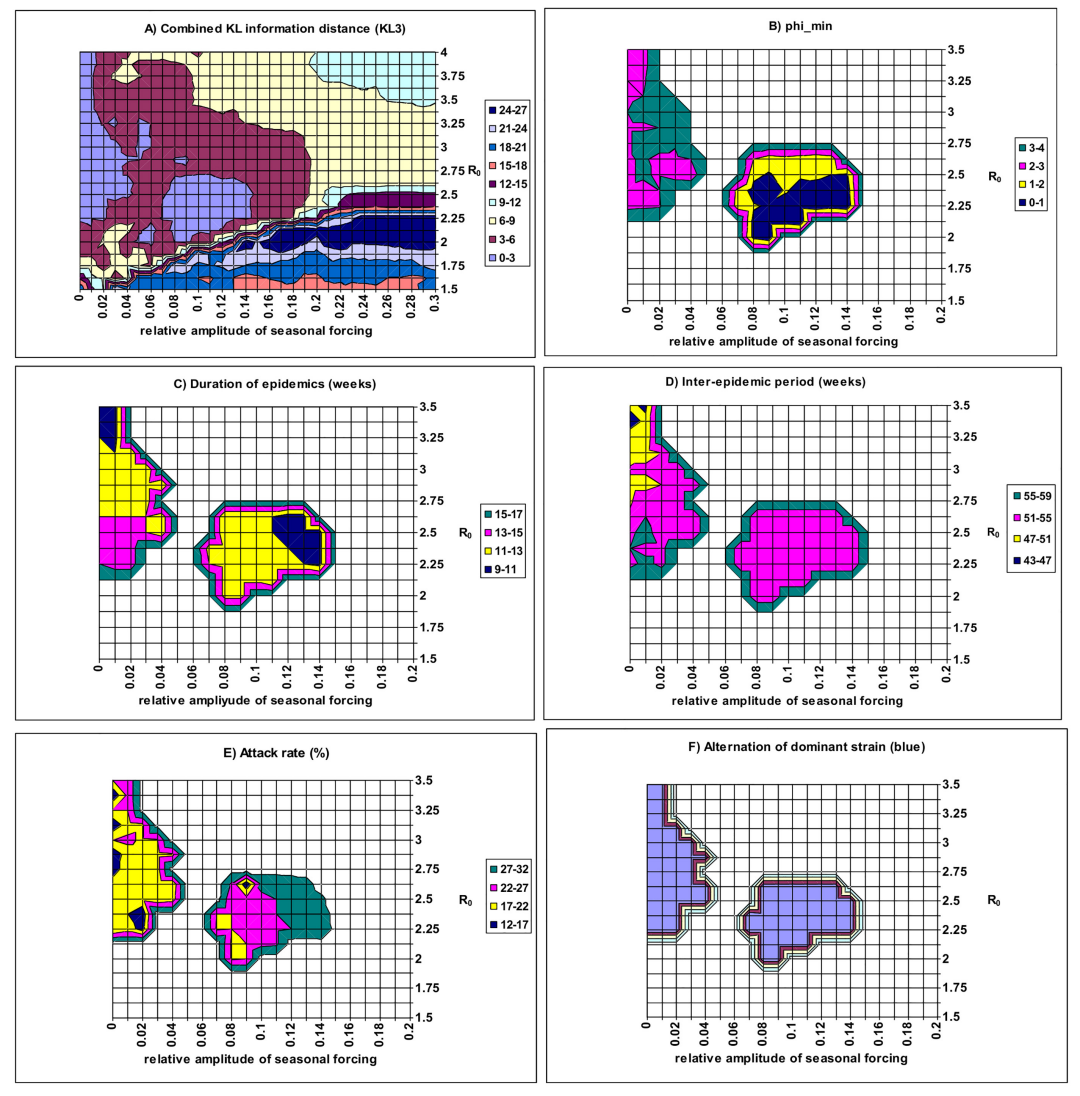
A) Model fit as a function of the basic reproductive number (*R*_0_) and the relative amplitude of seasonal forcing (*δ*) to illustrate the impact of seasonal forcing in the transmission rate. The co-transmission rate is assumed as *β*_d_ = *β/*4 and other parameters have the baseline values in Table 2. The best value for *ϕ* was searched on the interval [0,6] for each pair of (*R*_0_, *δ*). B) shows the values of *ϕ* for which the minima of the combined KL information distance (KL3) (i.e., the best–fitting) are found. C)-F) show the corresponding characteristic features of the epidemics for the minima of KL3. The two best fitting regions with KL3<3.0 emerge and correspond to two different mechanisms for epidemic cycling: intrinsic and extrinsic. To make their characteristics to be easily identified, only the relevant values are shown in panels 8B, 8C, 8D, 8E and 8F.

Finally, we explore the situation that includes seasonal forcing in the transmission rate as described in Eq (4). The results are shown in Fig 8. The effect of *δ* on KL3 depends on the values of *R*_0_. When *R*_0_ is large, KL3 will increase monotonically with the relative amplitude *δ*. However, when *R*_0_ is small, KL3 is not clearly associated with the relative amplitude *δ*. As the KL3 values are larger when *δ* >0.3 than that when *δ* <0.3, Fig 8A only displays the situation for *δ* < 0.3. With the inclusion of sufficient seasonal forcing in the transmission rate, the frequency of epidemics is determined by its yearly variation (Fig 8D; cf. [14]). According to our tentative criterion of KL3<9.0, the model outputs from a wide region of model parameters (e.g., *R*_0_ >1.7 and low values of *δ*) resemble the typical patterns of seasonal flu. Two regions have KL3 <3.0: one is the region of *δ* ~0.0 and *R*_0_ >2.2, with *ϕ* ranging from 2.0 to 4.0; the other is the region of *δ* = 7–15% and *R*_0_ = 2.0–2.7 with *ϕ* around 1.0 (Fig 8A and 8B). They correspond to two different mechanisms for epidemic cycling that we call for convenience the intrinsic and extrinsic mechanism regions. All these results are obtained under the assumption of the co-transmission rate *β*_d_ = 0.25*β*. For the situation of no co-transmission (i.e., *β*_d_ = 0), we obtained similar distributions of KL3 and the other characteristic features, except for the required values of *ϕ* increasing to 3.0–5.0 within the intrinsic mechanism region (see S2 File). This increase in *ϕ* is in agreement with the results illustrated in Fig 7A (c.f. Fig 4A of [16]). Within these two regions, the model can well generate the observed infection time series (Fig 8C–8E); and the dominant strain alternates among the three strains between epidemics (Fig 8E). This suggests that though seasonal forcing can facilitate the generation of empirical patterns of seasonal flu, co-transmission and infectivity enhancement surely provide another mechanism that can generate the empirical patterns of seasonal influenza.

## Discussion

The most obvious explanation for seasonal patterns of influenza is the variation in transmission rate caused by yearly environmental changes. In this study, we show an alternative mechanism for it. Our investigations show that the combination of both cross-immunity during sequential infection and infectivity enhancement within concurrent infection can generate the empirical patterns of both the periodic incidence and the alternation of the dominant strain in seasonal influenza. Within the accepted ranges of other transmission parameters, our model outputs closely resemble the empirical patterns of epidemic cycling when cross-immunity ranges from 0.5 to 0.8 and both co-transmission rate and infectivity enhancement are at intermediate levels.

Seasonal influenza viruses circulate worldwide and cause annual epidemics. To prevent and control flu, it is important to understand the underlying mechanisms of the annual patterns. Over the years much effort has been made to analyse and model seasonal influenza (e.g., [12, 14, 15, 18, 24, 25, 64, 68, 69, 70, 71, 72, 73, 74, 75, 76, 77]). Overall, these studies propose an extrinsic mechanism caused by environmental factors [10, 11, 73]. This appears obvious because transmission of influenza viruses depends on a number of environmental conditions especially climatic conditions and human gathering behaviours [70, 71, 72, 73, 74, 75, 76, 77]. Seasonal influenza is caused by different influenza viruses such as A/H1N1 and A/H3N2 and subtype B, and the dominant strain also cycles between epidemics [5, 6, 19]. However, a pure extrinsic mechanism cannot explain this [14, 64].

Concurrent infection [32, 33, 44, 45, 46, 47, 48, 49, 50, 51, 52, 53, 54, 55, 56, 57, 58, 59, 60] and sequential infection among co-circulating influenza viruses occur. Although the cross-immunity during sequential infection is well known, proof for the existence of interactions between strains within concurrent infection is lacking. To our knowledge, there are some observations and theoretical reasoning for their existence [40, 60, 61] and some indirect measurements of their possible values [43, 62]. In this study we explore an alternative explanation for both periodicities in incidence and alternation of the dominant strain based on strain interactions. Non-linear models with enhanced transmissibility are well known for generating complex bifurcation structure and periodic behaviour [16, 78]. In this study we concentrate on the cyclical behaviours by skipping the complex bifurcation details. We simply estimate the pattern of the time series by averaging over a long period (1000 epidemics after a burn-in period of 20000 years). We noticed that when both co-transmission rate (*β*_d_) and infectivity enhancement within concurrent infection (*ϕ*) are low, only endemics with constant incidence will be generated (Fig 7). In the absence of co-transmission, generating the empirical patterns of seasonal flu requires a moderate value of infectivity enhancement *ϕ* (about 3 in the situation illustrated in Fig 7). With the presence of co-transmission, the requirement for *ϕ* is reduced further. That is, infectivity enhancement within concurrent infection could be compensated by co-transmission to produce the empirical patterns (also see Fig 4A of [16]).

The crucial question arising from our modelling study is: what are the magnitudes of the co-transmission rate (*β*_d_) and the infectivity enhancement (*ϕ*) required to produce the observed patterns of seasonal influenza? Following Truscott et al. [14], the typical seasonal influenza epidemic was assumed to have an outbreak duration of 11 weeks and an annual attack rate of 15% and an epidemic period of one year. The Kullback-Leibler information distance between predicted and empirical epidemics was used to find model parameters that generated the typical patterns observed. The best fitting values for *ϕ* may appear a bit high as shown here in the SIRS epidemic model of three strains. In the situation of *β*_d_ = 0, for example, the required value for *ϕ* could be greater than 3.0. However, Zhang and Cao [16] show that the requirement reduces when the number of co-circulating strains increases. This implies that under the circumstance of a large number of co-circulating strains the empirical patterns of seasonal flu can be recreated even if infectivity enhancement is not strong (i.e., *ϕ ~*1) (see Fig 4A of [16]). If defining different strains by different genotypes (cf. [18, 69]), there are a large number of strains. Even if we define strains by their serological characteristics, the number of different influenza virus strains should be much larger than three [70].

To test which mechanism is more likely, extrinsic or intrinsic, a seasonal variation in the transmission rate was introduced into the model (see Eq (4)). Model fitting exercises show two best fitting regions (see Fig 8, also S2 File), which suggests two possible mechanisms for epidemic cycling. The intrinsic mechanism region is the long band having the relative amplitude *δ* ≈0 but requiring infectivity enhancement; the extrinsic mechanism region is the round island which includes seasonal forcing but requires no infectivity enhancement. As far as the combined Kullback-Leibler information distance is concerned, it is not possible to distinguish which mechanism is better or more parsimonious.

It is interesting to compare our model with other extrinsic models for epidemic cycles in both incidence and strain. Truscott et al. [14] found that in an age-structured population and when there are two strains that are affected by seasonal forcing, a weak cross-immunity (0.3–0.5) is necessary to recreate observed patterns in flu time-series data. Koelle et al. [4] examined a two strain cholera model using an estimate of the time varying reproductive rate and found that the observed serotype cycles in cholera epidemics can be explained when the cross-immunity exceeds 95%. White et al. [3] also examined a two strain SIRS model for transmission dynamics of groups A and B human respiratory syncytial virus in England & Wales and Finland. They found that the change in the dominant group can be explained by a 65% reduction in the susceptibility to (and the infectiousness of) secondary homologous infections and a 16% reduction in the susceptibility to (and the infectiousness of) secondary heterologous infections. These models consider seasonal forcing and cross-immunity, but don’t include the strain interactions within concurrent infection. These models require a relatively high level of relative amplitude in seasonal forcing to explain the empirical patterns in seasonal flu (about 15–30%), in cholera (about 30%) and in respiratory syncytial virus infection in humans (about 35% for Finland and about 82% for England & Wales). Nevertheless, the basic understanding of those studies is that recurrent epidemics are induced by seasonal forcing while alternation in the dominant strain between epidemics is due to the negative association created by cross-immunity. Our model, which includes strain interactions within concurrent infection but ignores seasonal forcing, can also explain the empirical patterns seen in seasonal flu when cross-immunity is 50–80%. When relaxing the assumption of a constant transmission rate, the relative amplitude in seasonal forcing required in our model (Fig 8) is 7–14%, which is lower than that required in [14].

Recker et al. [7] considered a four strain SIR model by decomposing the antibody dependent enhancement during sequential infection into two aspects: increased susceptibility to secondary infections and increased transmissibility from individuals suffering secondary infections. They showed that both the observed temporal patterns and the replacement in the dominant serotype of dengue can be reproduced without the need for extrinsic factors such as seasonal forcing or stochasticity. Hence it was suggested that it is the enhancement in both susceptibility and transmission during the sequential infection that induces irregular patterns of dengue. Nevertheless, this mechanism does not rule out cross-immunity which could be a contributing factor for the asynchronicity of strains [79]. As in [3, 4, 14], Recker et al. [7] did not include in their model the existence of concurrent infection and any strain interactions within it. However, both Recker et al. [7] and our study demonstrate that in the absence of seasonal forcing, at least two different types of strain interaction are required to generate epidemic cycling. Antigenically diverse pathogens are common and they are a big challenge for modern medicine. Co-circulations of different strains of other multi-strain pathogens have been observed (e.g., [1, 2, 3, 4, 7, 8, 17]); concurrent infection and hence strain interactions within it are also possible (e.g. [80]). The current lack of evidence for them may be due to our lack of intention or tool to search for them. This study shows that the key mechanism for the existence of epidemic cycling may lie in interactions between strains within concurrent infection and during sequential infection. Therefore, the intrinsic mechanism we show here for seasonal flu may also apply to other infectious diseases caused by antigenically diverse pathogens.

For simplicity, we have ignored the heterogeneity among age groups. However, Truscott et al. [14] identified age-structure as a necessary factor for recreating the patterns seen in time series of seasonal flu. Both Fig 5 of this study and Fig 3 of [14] explore the model behaviour as a function of transmissibility and duration of immunity. But the best fits are quite different: in [14] the best fit region is located in a narrow diagonal band while in this study it is located in a broad triangle. The former shows a highly restrictive relationship between transmissibility and duration of immunity while the latter suggests a weak relationship. These discrepancies may result from different assumptions made about age-structure and seasonal forcing in these models. Age-structure allows for examinations of the impact of non-random mixing patterns and heterogeneity in the infectivity and the susceptibility of different age groups. Inclusion of such heterogeneity will surely improve our model. Further we assume the same epidemiological characteristics for each of the three strains in our model (i.e. that the three strains are phenotypically indistinguishable). This is surely a simplification of the truth as the epidemiological characters of influenza A and B are different. A deterministic model was used to describe influenza epidemics over a long term in spite of there being evidence for strong stochastic behaviour during the inter-epidemic periods. To reflect the antigenic drift within each strain (lineage), infection-induced immunity is assumed to wane, as a result of immune escapement and loss, and each strain was regarded as a constant biological identity. In this study, the same waning rate of immunity (equivalent to the rate of antigenic drift) is assumed for each strain. A recent comprehensive study [69] of strains co-circulating in the human population shows that the rates of antigenic drift are different among them with A/H3N2 the fastest and B strain the slowest. Heterogeneity in the intrinsic factors of strains and the stochastic nature of the transmission process will also make the model more realistic [7, 70]. All of these will be considered in future work.

## Supporting Information

S1 Fig Diagram of epidemic scenarios within the plane of cross-immunity (*ψ*) and infectivtity enhancement (*ϕ*). (TIFF)

S2 Fig Bifurcation from endemics with constant incidence to cyclical or chaotic epidemics when cross-immunity (*ψ*) is 0.65. (TIFF)

S3 Fig Times series of infections at different values of the infectivity enhancement (*ϕ*) shown in S2 Fig. (TIFF)

S4 Fig A variant of Fig 4 under *β*_d_ = 0, *ε =* 50, and *δ =* 0. (TIFF)

S5 Fig A variant of Fig 4 under *β*_d_ = *β*/3, *ε =* 150, and *δ =* 0. (TIFF)

S6 Fig A variant of Fig 8 under *β*_d_ = 0, *d*_I_ = 2.0 days, *ε* = 150. (TIFF)

S7 Fig A variant of Fig 8 under *β*_d_ = *β*/3, *d*_I_ = 2.0 days and *ε* = 50. (TIFF)

S8 Fig A variant of Fig 8A under *β*_d_ = *β*/4, *d*_I_ = 3.0 days and *ε* = 50. (TIFF)

S9 Fig A variant of Fig 8A under *β*_d_ = *β*/4, *d*_I_ = 4.0 days and *ε* = 150. (TIFF)

S1 File Strain interactions and bifurcation into cyclical epidemics. (DOCX)

S2 File More explorations of the model parameters. (DOCX)
