# Supplementary figures and images for: Strain Interactions as a Mechanism for Dominant Strain Alternation and Incidence Oscillation in Infectious Diseases: Seasonal Influenza as a Case Study
Source: PLoS One. 2015 Nov 12;10(11):e0142170. doi: 10.1371/journal.pone.0142170 (PMC4642928; doi:10.1371/journal.pone.0142170)

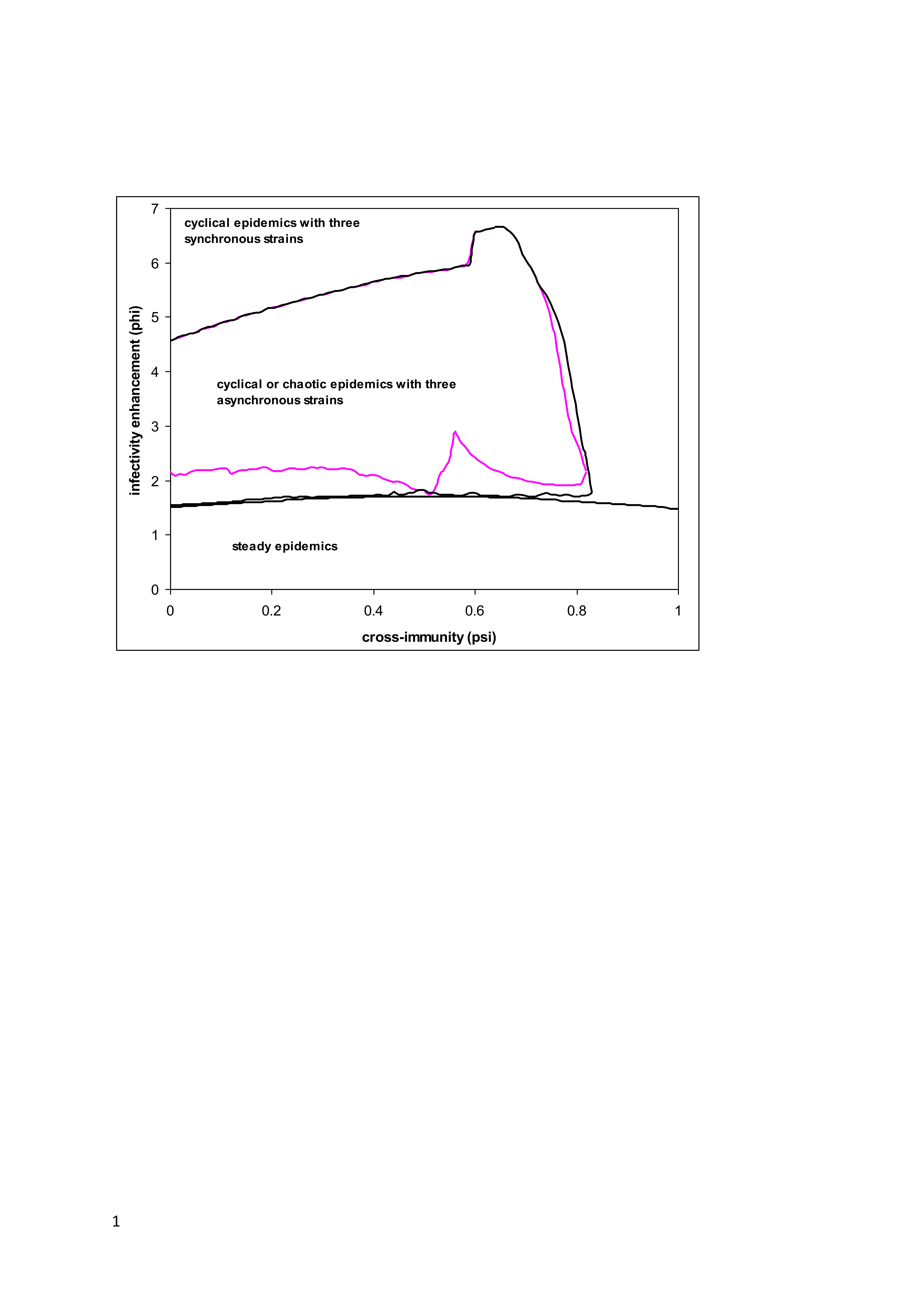

Supplement: S1 Fig — (TIFF) [file pone.0142170.s001.tiff]

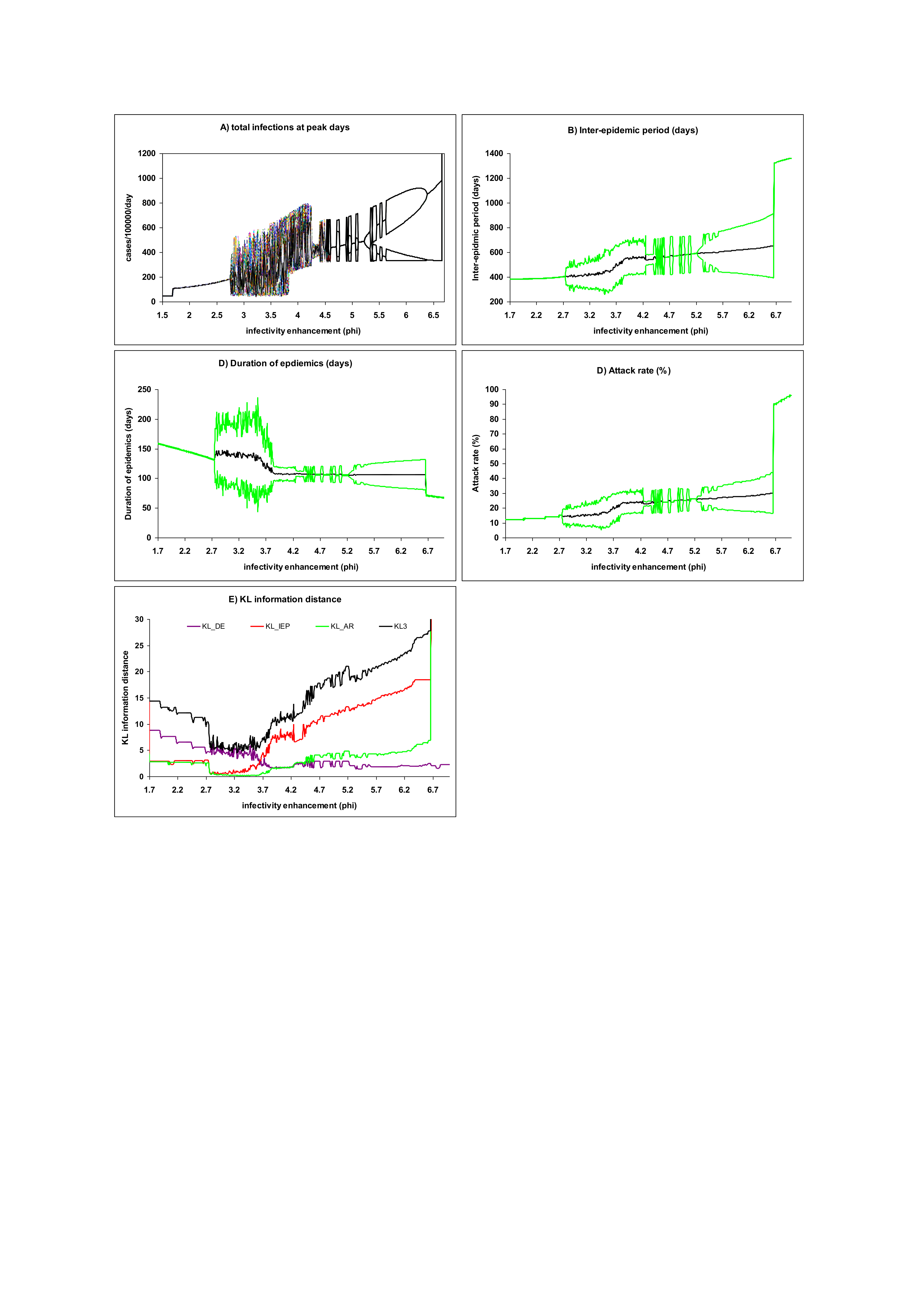

Supplement: S2 Fig — (TIFF) [file pone.0142170.s002.tiff]

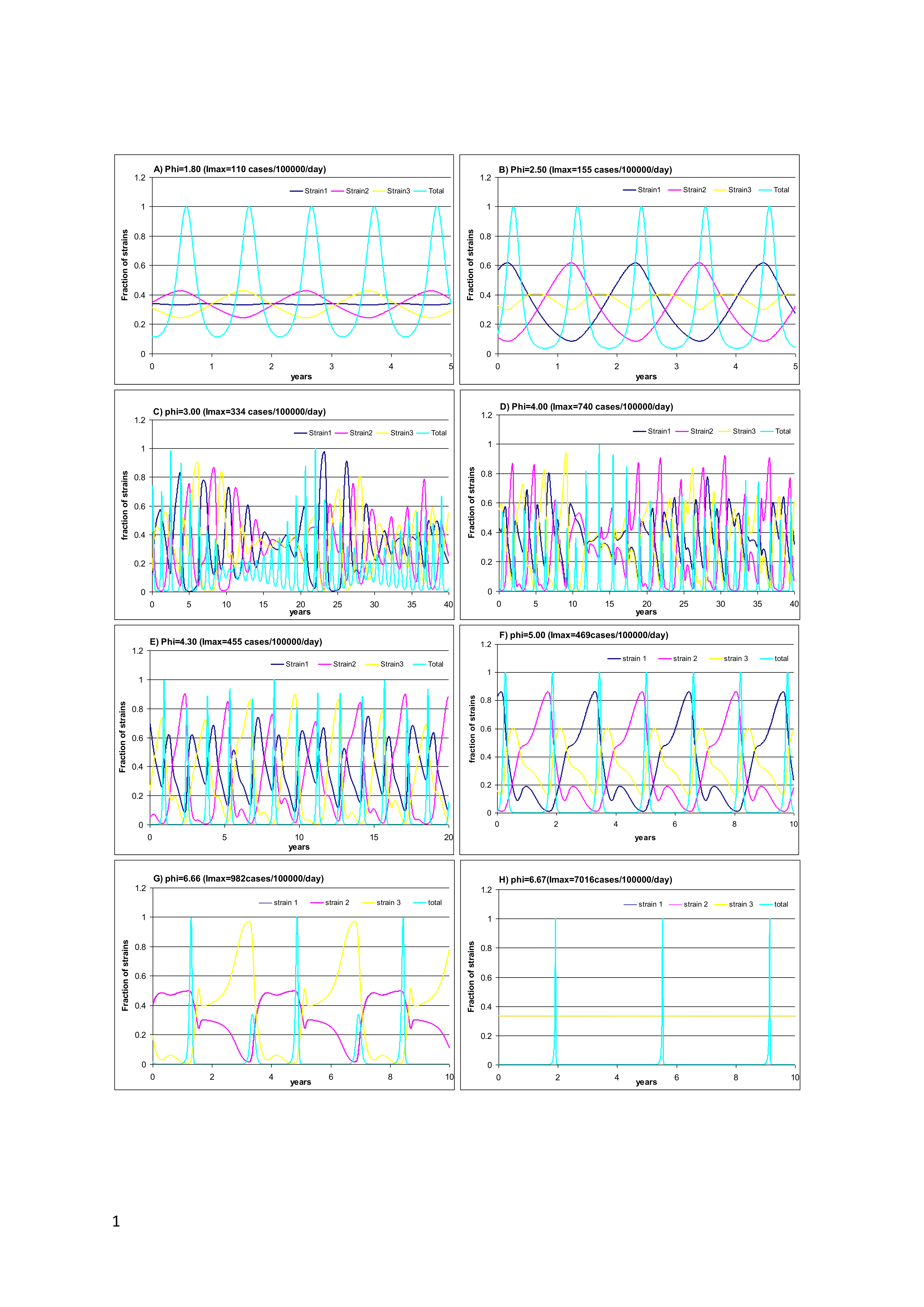

Supplement: S3 Fig — (TIFF) [file pone.0142170.s003.tiff]

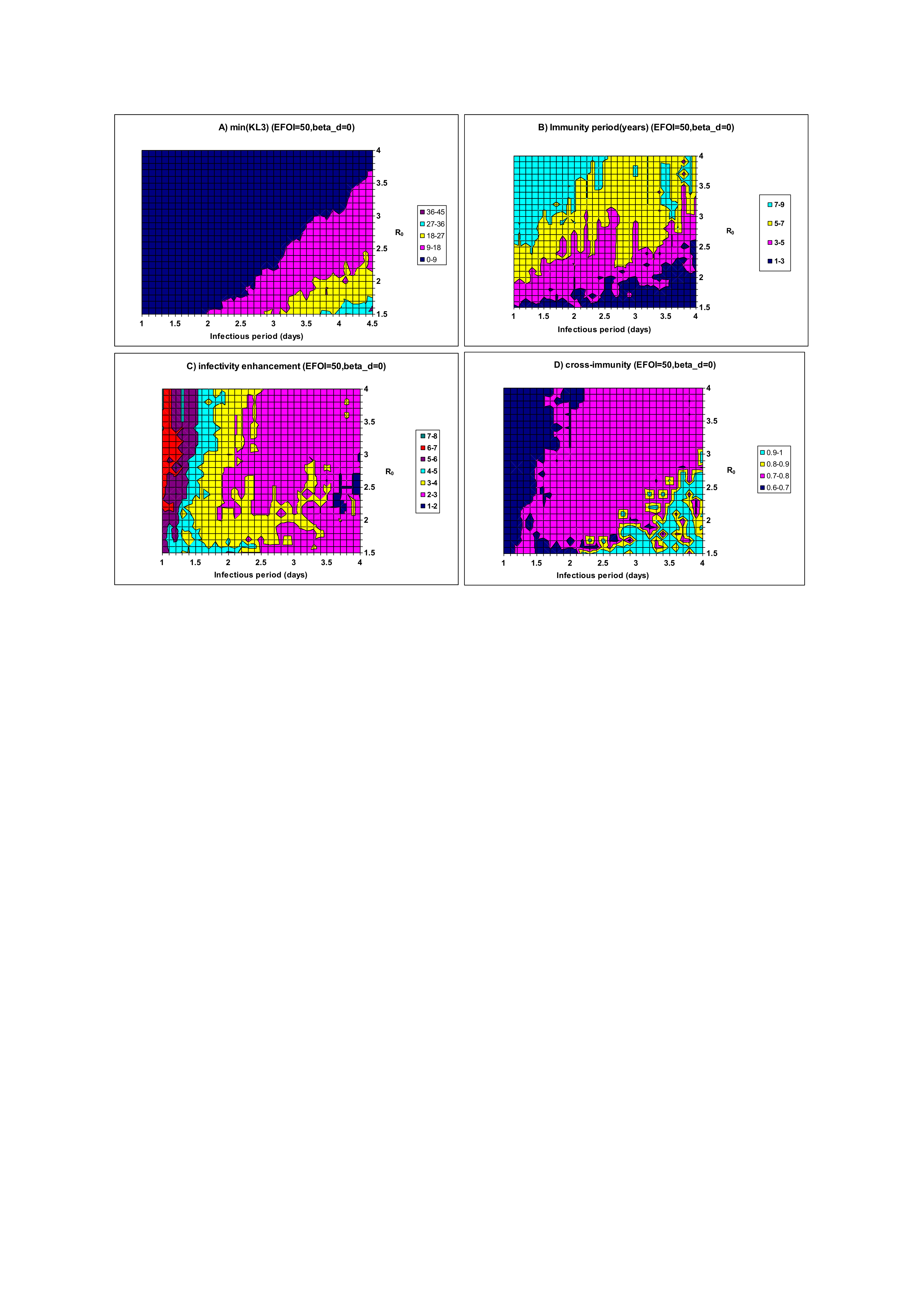

Supplement: S4 Fig — (TIFF) [file pone.0142170.s004.tiff]

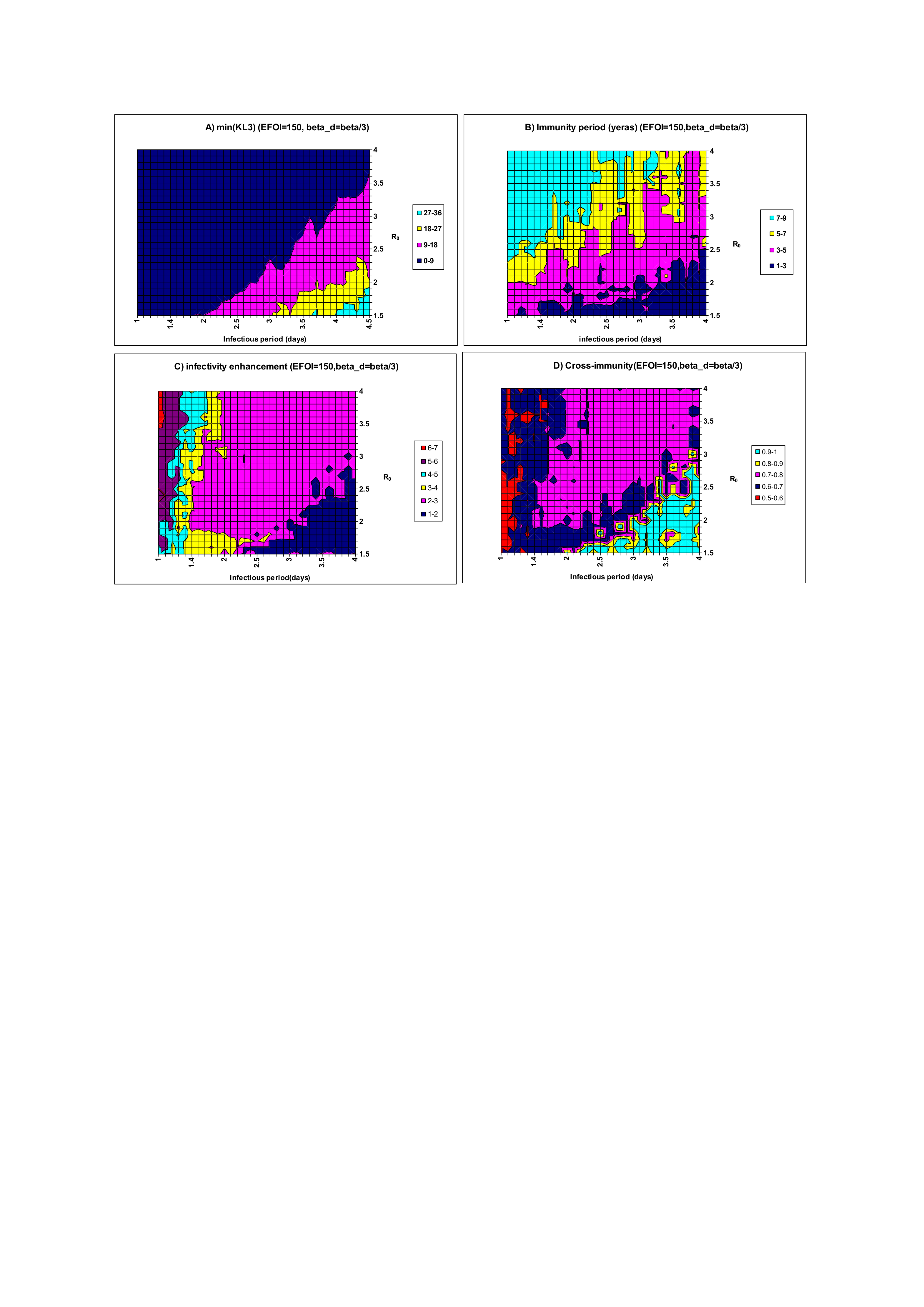

Supplement: S5 Fig — (TIFF) [file pone.0142170.s005.tiff]

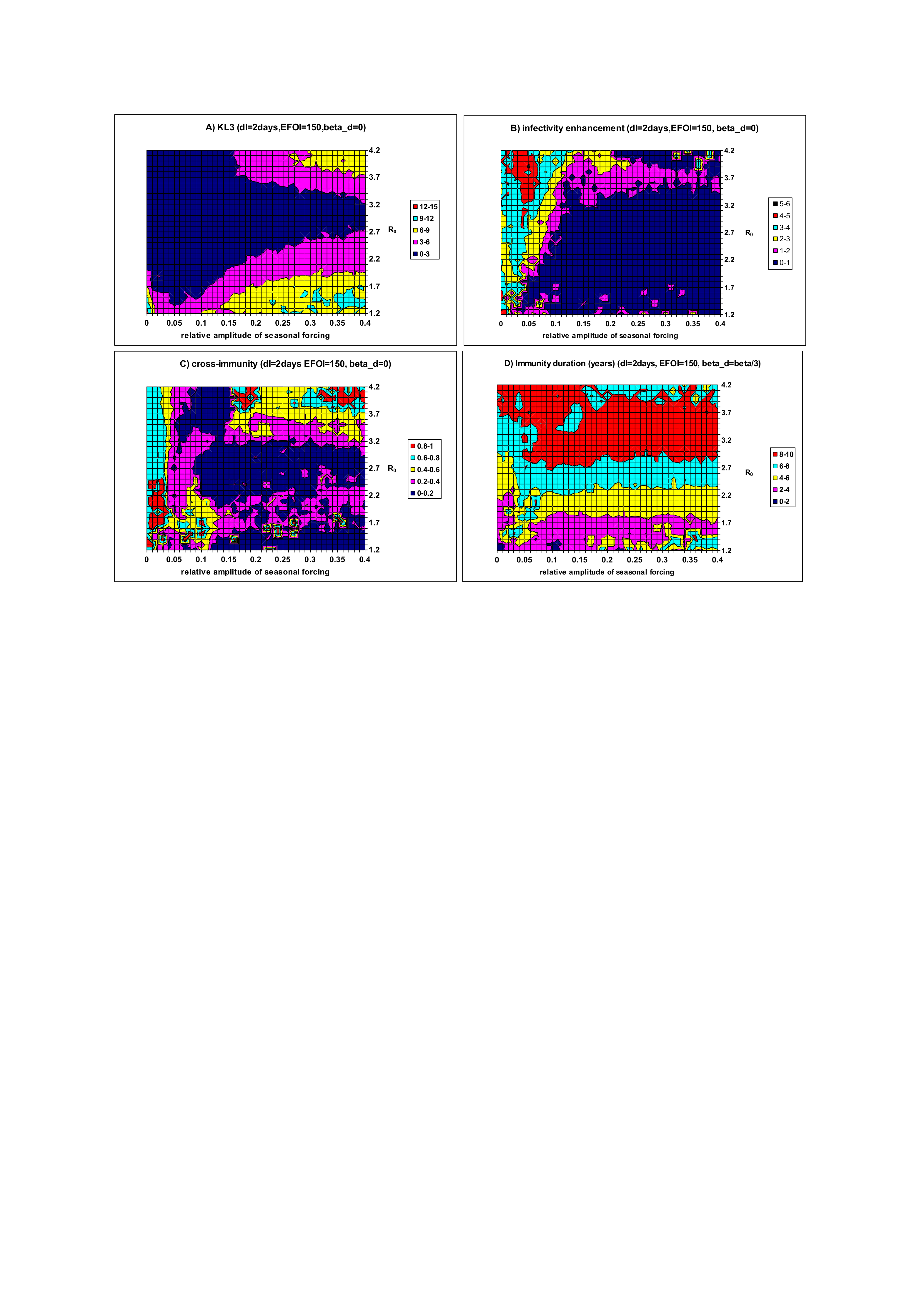

Supplement: S6 Fig — (TIFF) [file pone.0142170.s006.tiff]

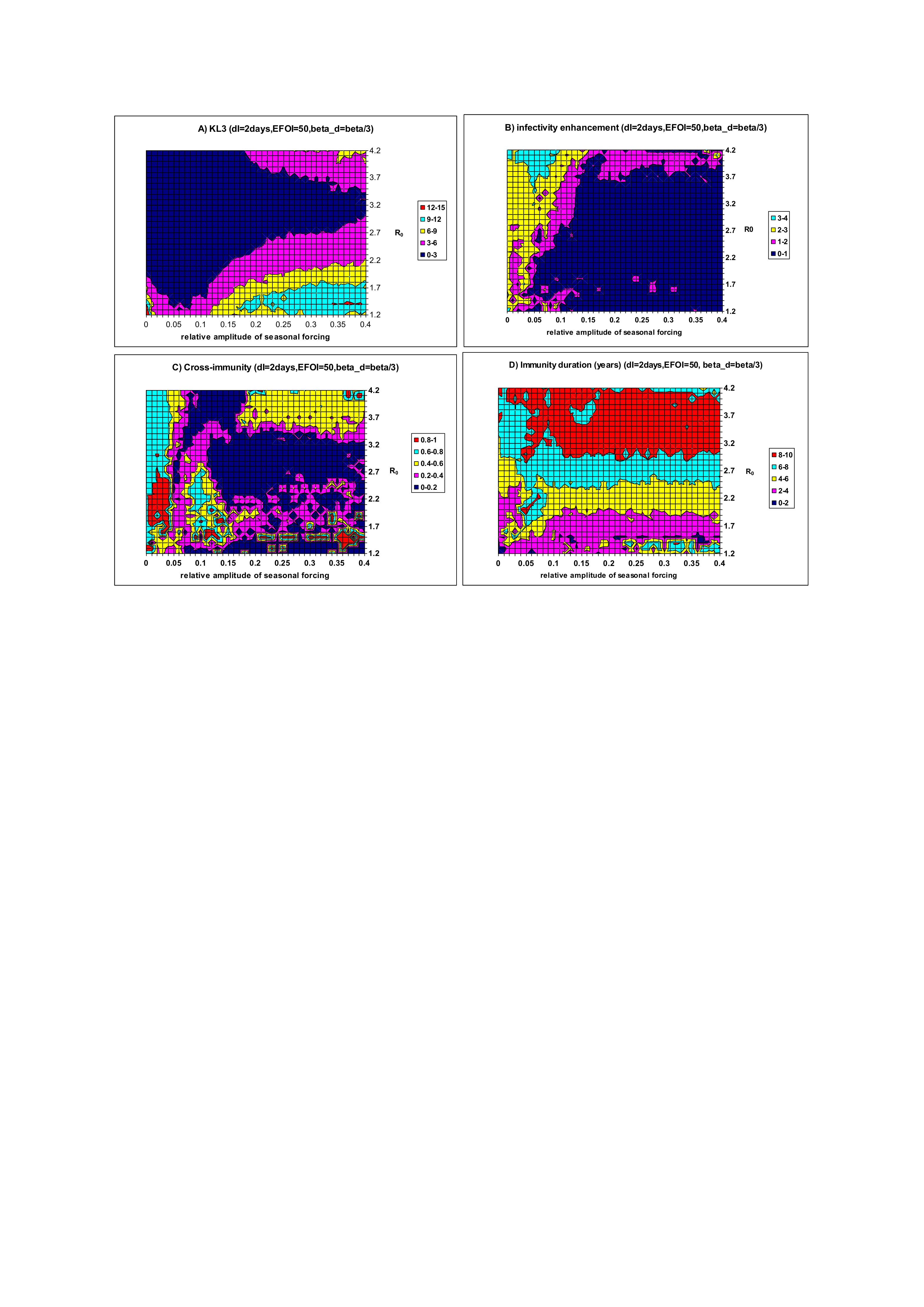

Supplement: S7 Fig — (TIFF) [file pone.0142170.s007.tiff]

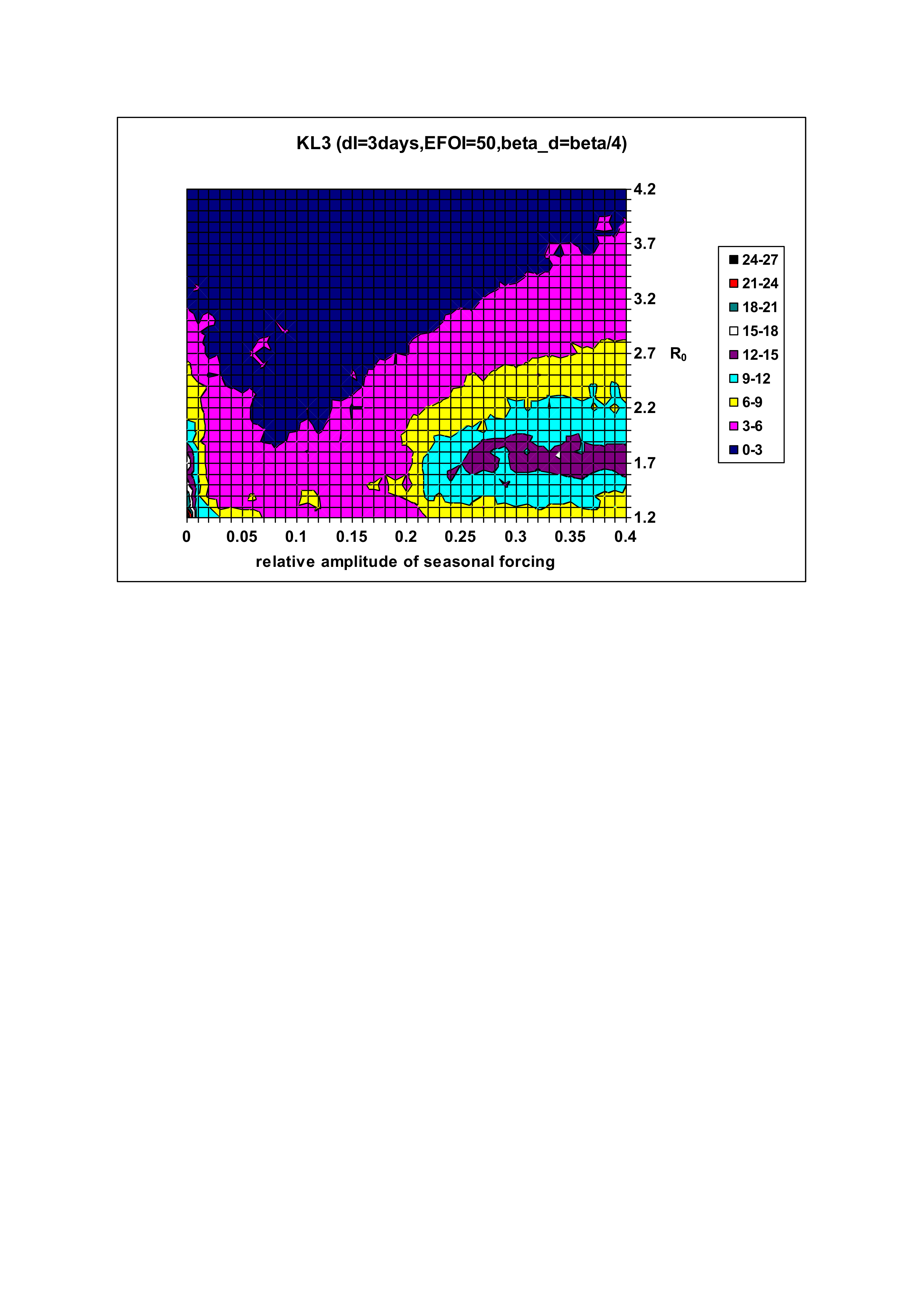

Supplement: S8 Fig — (TIFF) [file pone.0142170.s008.tiff]

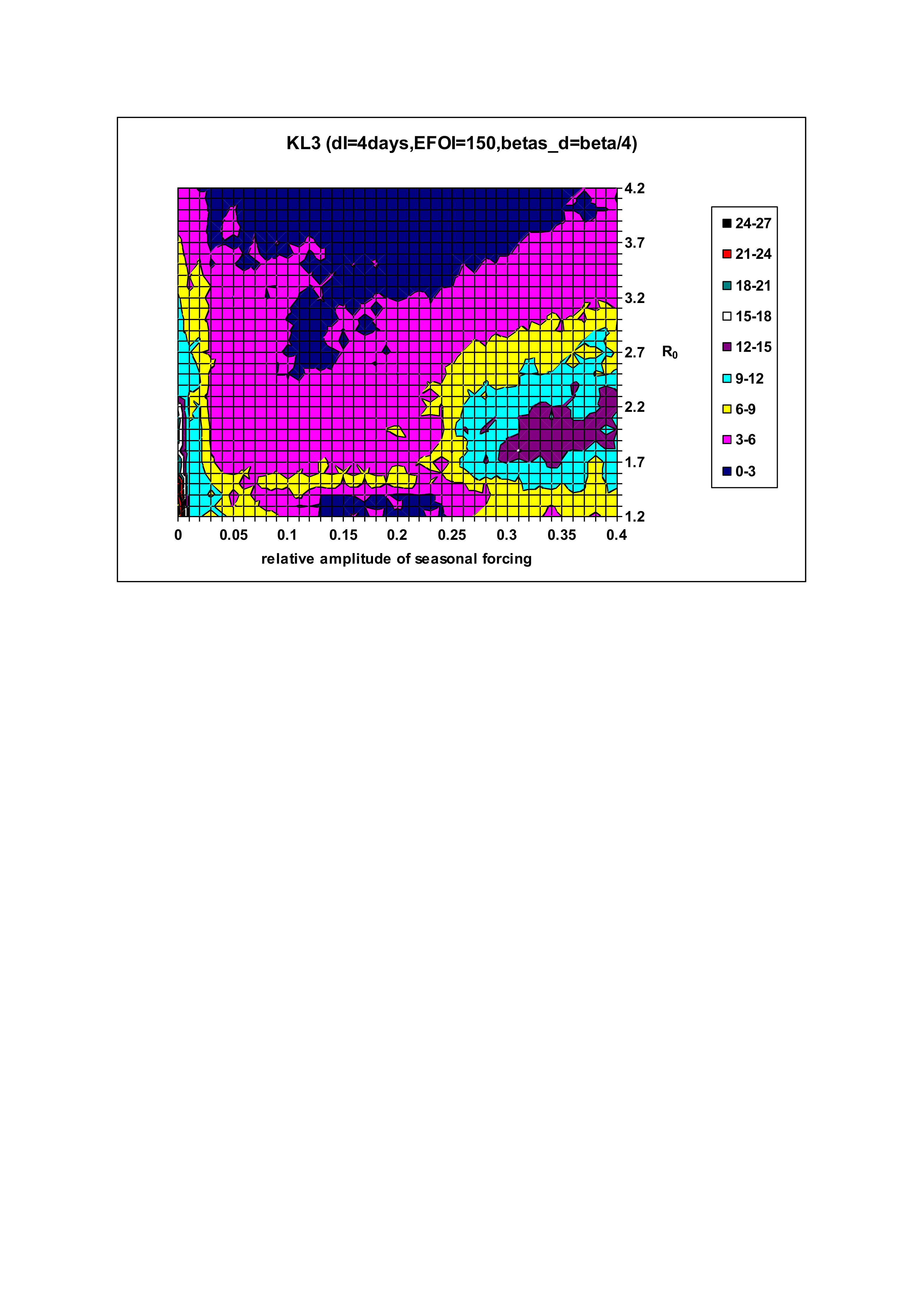

Supplement: S9 Fig — (TIFF) [file pone.0142170.s009.tiff]
