# Supplementary material for: Strain Interactions as a Mechanism for Dominant Strain Alternation and Incidence Oscillation in Infectious Diseases: Seasonal Influenza as a Case Study
Source: PLoS One. 2015 Nov 12;10(11):e0142170. doi: 10.1371/journal.pone.0142170 (PMC4642928; doi:10.1371/journal.pone.0142170)
Supplement: S1 File — (DOCX) [file pone.0142170.s010.docx]

**Supporting information 1:** Strain interactions and bifurcation into cyclical epidemics

In this Supporting Information we show that strain interactions generate cyclical or chaotic epidemics in the absence of external seasonal forcing in the transmission rate. The initial number of each type of infection is randomly selected and a burn-in period of 20000 years is allowed for the three strains to spread and to fully interact, the dynamical patterns are then analysed from a further 200 epidemics. We only consider how cross-immunity and infectivity enhancement interact to affect the generation of cyclical or chaotic epidemics while all the other model parameters are fixed at the baseline values in Table 2.

Only steady epidemics are possible if infectivity enhancement (*φ*) is weak (<1.5 in the situation of Figure S1). When *φ* exceeds a threshold value, steady epidemics bifurcate into cyclical ones. With strong cross-immunity (*ψ*>84%), only cyclical epidemics are generated and strains remain synchronous; otherwise, strains quickly become asynchronous. When *φ* increases further chaotic epidemics emerge and alternation in the dominant strain occurs between epidemics. Nevetheless, as infectivity enhancement becomes even stronger, epidemics become cyclical again and the three strains return to becoming synchronous. Note that the dominant strain disappears at exactly the same value of *φ* where the three strains synchronise. Detailed dynamic patterns are illustrated in Figure S2 and S3.

Figure S2 shows the dynamic patterns and their summary characteristics when cross-immunity *ψ* =0.65. Cyclical epidemics emerge at *φ* =1.70 and chaotic epidemics burst out if *φ* =2.75 to 4.47, after which some multi-period cyclical epidemics emerge intermittently along with the occurrence of simple period-1 epidemics. The three strains become asynchronous once the total incidence bifurcates into a cyclical pattern when *φ* > 1.70. Alternation in the dominant strain occurs when *φ* exceeds 2.14 (Figure S1). However, when *φ* exceeds 6.66 (Figure S3H), the three strains synchronise although the total incidence oscillates with period-1. The inter-epidemic period sharply increases to 1320 days (longer than 3.5 years) and the duration of epidemics shortens to 70 days, while the attack rate during an epidemic shoots up to over 90%. Some infection time series are showed in Figure S3.

We notice that as *φ* increases from 1.70 to 6.66, the average inter-epidemic period increases from about one year to 1.8 years, but sharply doubles to over 3.5 years when *φ* ≥ 6.67 (Figure S2B). Similar changes also occur in the attack rate: when *φ* increases from 1.7 to 6.66, the average attack rate increases from 12% to 30%; after *φ* =6.67, it shoots up to over 90% (Figure S2D). Here we avoid the term "annual attack rate" because the inter-epidemic period is larger than one year when infectivity enhancement is strong. The average duration of epidemics decreases from about 150 days to 100 days, and it drops to 70 days when *φ ≥*6.67 (Figure S2C). Figure S2E shows that the epidemics generated from the model with 2.74≤*φ≤*4.41 (i.e., the chaotic region) best resemble the typical patterns of observed seasonal flu time series data.

Figure S3 shows the time series of the cyclical and chaotic epidemics shown in Figure S2A. When *φ* just exceeds 1.70, the total incidence oscillates while only two strains become asynchronous and the third remains nearly constant (Figure S3A). When *φ* > 2.14, more than half of infections are caused by one strain (i.e., the dominant strain emerges) and alternation in the dominant strain among the two strains emerges (Figure S3B). The third strain oscillates at low levels if *φ* is less than 2.75. If *φ* > 2.75, epidemics turn chaotic and the dominant strain changes among the three strains. As *φ* continues to increase to 4.41, regular periodic epidemics appear again. When *φ* is larger than 6.66, incidence oscillates with period-1 and the three strains synchronise.

It is interesting to note that when there is no cross-immunity (*ψ*=0), the three strains can become asynchronous when 1.54<*φ*<4.56, and the dominant strain among the three strains can change between epidemics if 2.16<*φ* <4.56 (Figure S1). This observation is in contrast with the conventional thinking: dominant strain changes are due to the negative association between strains generated by cross-immunity (Truscott et al 2012). However, when setting EFOI (ε) =0 and allowing two strains to co-circulate within the population, the calculations show that only synchronous strains are possible when there is no cross-immunity (*ψ*=0) which is in agreement with the conventional thinking (Truscott et al 2012).

**Figure legends**

Figure S1 Diagram of dynamic scenarios within the plane of cross-immunity (*ψ*) and infectivtity enhancement (*φ*). Other model parameters take the baseline values in Table 2. The plane is divided into three regions: (i) steady epidemics where *φ* is low, (ii) cyclical or chaotic epidemics with three asynchronous strains and (iii) cyclical epidemics with three synchronous strains. The red curve encloses the region where the dominant strain alternates between epidmics with the dominant strain defined as the strain responsible for more than 50% of all infections during an epidemic (as in the main text).

Figure S2 Bifurcation from steady epidemics to cyclical or chaotic epidemics when infectivity enhancement (*φ*) increases. Cross-immunity (*ψ*) is 0.65 and other parameters take the baseline values in Table 2. A) The daily number of infections at peak days during epidemics expressed as cases per 100000 persons per day, B) the inter-epidemic period (the black solid line represents the average of the values and the green thin lines represent the 95% range of the values), C) duration of epidemics (the meaning of the black solid line and the green thin lines are the same as in Figure S2B), D) attack rate (the meaning of the black solid line and the green thin lines are the same as in Figure S2B) and E) the KL information distance to the typical patterns of seasonal flu time series data. Note that when *φ* passes the critical value of 6.67, the dynamic system changes dramatically: epidemics become of period-1 while the daily number of infections at peak days exceeds 7000 cases per 100000 persons per day (not shown in panel A) and there are sudden increases in both the inter-epidemic period and the attack rate but a drop in the duration of epidemics.

Figure S3 Times-series of infections at different values of infectivity enhancement (*φ*) shown in Figure S2. Here we show the total daily number of infections relative to its maximum value (*I*_max_), and the fractions of the three strains. A) *φ* =1.80 with a maximum daily number of infections (*I*_max_) of 110 per 100000 persons, B) *φ* =2.50 with *I*_max_ =155, C) *φ* =3.00 with *I*_max_ =334, D) *φ* =4.00 with *I*_max_ =740, E) *φ* =4.30 with *I*_max_ =455, F) 5.00 with *I*_max_ =469, G) *φ* =6.66 with *I*_max_ =982 and H) *φ* =6.67 with *I*_max_ =7016.
