# Supplementary material for: Strain Interactions as a Mechanism for Dominant Strain Alternation and Incidence Oscillation in Infectious Diseases: Seasonal Influenza as a Case Study
Source: PLoS One. 2015 Nov 12;10(11):e0142170. doi: 10.1371/journal.pone.0142170 (PMC4642928; doi:10.1371/journal.pone.0142170)
Supplement: S2 File — (DOCX) [file pone.0142170.s011.docx]

**Supporting information 2:** More explorations of the model parameters

In this supporting information, we will show more explorations in model parameter space. There are eight parameters that can be allowed to vary: infectious period (*d*_I_), transmissibility (*β, equivalently R*_0_ =*βd*_I_), infectivity enhancement (*φ*), cross-immunity (*ψ*) and immunity duration (*D*), co-transmission rate (*β*_d_), the external force of infection (EFOI,*ε*) and relative amplitude (*δ*) of seasonal forcing. In principle we should explore model dynamic behaviour over 8-dimension space. This is surely a huge amount of calculations! To show the evidence that conclusions drawn in the main text are robust, we only illustrate more explorations for the situations of Figure 4 and 8 as examples.

**Figure 4**

In the text, Figure 4 shows the best fitting results for the epidemic system under the values of five parameters: *d*_I_, *R*_0_, *φ*, *ψ, D* while *β*_d_, *ε* and *δ* were fixed at the baseline value listed in Table 2: *β*_d_ = *β*/4, *ε =*94*, δ =*0*.* Here *ε* is expressed as the number of infections introduced per year over the whole population of size *N* =6.3e+7. We further give results under two other situations: *β*_d_ =0, ε *=*50*,* and *δ =*0 (Figure 4S) and *β*_d_ = *β*/3, ε *=*150*,* and *δ =*0 (Figure 5S). Under these two situations, the time series of infection that most closely resemble the empirical patterns are generated under the parameter regions: cross-immunity (*ψ*) from 0.5 to 0.8, immunity duration (*D*) from 4 to 9 years*,* and infectivity enhancement from 2 to 7, which are similar to the results from Figure 4 in text. This suggests that our conclusions drawn from Figure 4 should hold under wider range of model parameters than the assumed baseline values in Table 2.

**Figure 8**

In the text, Figure 8, which demonstrates how the epidemic dynamics change with *R*_0_ and *δ*, is obtained by fixing *β*_d_, *φ*, *ψ* and *D*, *d*_I_, and *ε* at the baseline values listed in Table 2. To exhaustively explore the parameter space, we search the values of three model parameters *φ*, *ψ* and *D* that minimize the KL3 while fixing values of parameters *β*_d_, *d*_I_=2.0 and *ε*. Four situations are shown: *β*_d_ =0, *d*_I_ =2.0 days and *ε* =150 (Figure S6), *β*_d_ =*β*/3, *d*_I_ =2.0 days and ε =50 (Figure S7), *β*_d_ =*β*/4, *d*_I_ =3.0 days and *ε* =50 (Figure S8), *β*_d_ =*β*/4, *d*_I_ =4.0 days and *ε* =150 (Figure S9). Here the EFOI (*ε*) is expressed as the number of infections introduced per year over the whole population of size *N* =6.3e+7. In the last two situations (Figures S8 and S9), only the combined KL distance (KL3) of the best fittings are shown. For all these situations, both the intrinsic mechanism due to strain interaction and the external mechanism due to seasonal forcing are equally working in view of the values of KL3. When *δ* =0, the best fitted epidemics require *φ* >2.9 (Figure S6B) or 2.0 (Figure S7B); when *δ* >0, *φ* reduces to around 1.0 (Figure S6B and S7B). When *δ* =0, the best fitted epidemics require *ψ* =0.60-0.8; when *δ* >0, *ψ* could be very weak (Figure S6C and S7C). Figures S8 and S9 show that with a long infection period (*d*_I_ >3 days) model fittings become worse. These figures indicate that when *β*_d_ < *β*/3, *d*_I_≤3 days, and *ε*<150 (i.e. 6.6×10^-9^ per individual per day), our conclusions drawn from Figures 8 still hold.

**Figure legends**

Figure S4 A variant of Figure 4 under *β*_d_ =0, *ε =*50*,* and *δ =*0*.*

Figure S5 A variant of Figure 4 under *β*_d_ = *β*/3, *ε =*150*,* and *δ =*0*.*

Figure S6 A variant of Figure 8 under *β*_d_ =0, *d*_I_ =2.0 days, *ε* =150.

Figure S7 A variant of Figure 8 under *β*_d_ =*β*/3, *d*_I_ =2.0 days and *ε* =50.

Figure S8 A variant of Figure 8A under *β*_d_ =*β*/4, *d*_I_ =3.0 days and *ε* =50.

Figure S9 A variant of Figure 8A under *β*_d_ =*β*/4, *d*_I_ =4.0 days and *ε* =150.
